# Mating system of free-ranging domestic dogs and its consequences for dog evolution

**DOI:** 10.1073/pnas.2421756122

**Published:** 2025-11-24

**Authors:** Clément Car, Roya Adavoudi, Andreas Berghänel, Melissa Vanderheyden, Andre E. Moura, Friederike Range, Giulia Cimarelli, Martina Lazzaroni, Rachel Dale, Ikhlass El Berbri, Gabriella J. Spatola, Timothy A. Mousseau, Sarah Marshall-Pescini, Małgorzata Pilot

**Affiliations:** ^a^Department of Evolutionary Genetics and Biosystematics, Faculty of Biology, University of Gdańsk, Gdańsk 80-308, Poland; ^b^Domestication Lab, Konrad Lorenz Institute of Ethology, Department of Interdisciplinary Life Sciences, University of Veterinary Medicine Vienna, Vienna 1160, Austria; ^c^Zoology and Animal Ecology Research Group, Department of Biology, University of Hildesheim, Hildesheim 31141, Germany; ^d^Behavioural Ecology Group, Wageningen University and Research, Wageningen 6708 WD, the Netherlands; ^e^Department of Chemistry, Life Science and Environmental Sustainability, University of Parma, Parma 43124, Italy; ^f^Department for Psychosomatic Medicine and Psychotherapy, University for Continuing Education Krems, Krems 3500, Austria; ^g^Department of Veterinary Pathology and Public Health, Agronomy and Veterinary Institute Hassan II, Rabat 10101, Morocco; ^h^Cancer Genetics and Comparative Genomics Branch, National Human Genome Research Institute, National Institutes of Health, Bethesda, MD 20892; ^i^Department of Biological Sciences, University of South Carolina, Columbia, SC 29208

**Keywords:** domestication, genealogy, mating system, reproductive success, social interactions

## Abstract

The polygamous mating system of free-ranging domestic dogs (FRDs) contrasts with the social monogamy typical of gray wolves and all other wild canids. The transition to polygamy in dogs could have been initiated by a shift from apex predator to human commensal during the early domestication stages. Here, we test this hypothesis by investigating the characteristics of the FRD mating system that could have been important for domestication. This inference is based on genome-wide Single Nucleotide Polymorphism (SNP) data from three geographically distinct populations, including behavioral data for one of them. The reconstructed genealogies form a wide network of kinship relationships resulting from the frequent occurrence of maternal and paternal half-siblings, reflecting male and female polygamy. Reproductive success is positively correlated with the strength of social interactions and the number of connections with opposite-sex individuals, implying a preference toward familiar mates and the importance of social relationships in determining mating patterns. This is supported by a nonrandom distribution of reproductive partners and a reproductive skew in males and females. Multiple paternity within litters points to female polygamy within a single estrus, and sexual size dimorphism implies sexual selection favoring larger males. Physiological changes resulting from polygamy, including increased male fertility and reduced breeding seasonality, could have facilitated the natural spread of novel adaptive traits and limited the introgression from wolves. The change in reproductive patterns, typically considered a consequence of selective breeding, could instead have occurred naturally in response to the dietary niche change, triggering further changes that facilitated the domestication process.

## Significance

How the wolf became domesticated by hunter-gatherer societies without prior experience of animal breeding is a long-standing puzzle. Differences in the behavioral ecology of wolves and dogs may provide important clues. In contrast to the prevailingly monogamous wild canids, free-ranging dogs (FRDs) are typically polygamous, suggesting that the transition to polygamy is associated with domestication. We studied the genetic mating system of three FRD populations and found consistent patterns of male and female polygamy, skewed reproductive success, and preference for familiar mates. Physiological and behavioral changes associated with the transition to polygamy may have facilitated the spread of adaptations to the anthropogenic niche without human intervention. This points to a key role of ecological and behavioral changes as domestication drivers.

Wild canids are typically classified as monogamous (1). However, in situations of high food abundance and/or dependence on more predictable anthropogenic food sources, a number of species experience shifts in their mating system from monogamy to polygamy (2–4). Accordingly, multiple breeding females were found in packs of gray wolves (*Canis lupus*) that were larger than usual due to population growth, intense hunting, or access to large prey (5–9). The occurrence of plural breeding in gray wolves varies between populations, and its maximum proportions have been estimated at 15% (8). Moreover, “sneaker” males, who produce offspring without bearing the costs of parenting, have been reported in some wolf populations, but this alternative strategy is maintained at low frequencies (5–7). Taken together, these findings show that although the predominant mating system of canids is monogamy, plasticity is present, with the species’ feeding ecology affecting the adoption of alternative mating strategies (1).

Becoming dependent on human-derived food is considered one of the main steps in the dog domestication process (10–12). The initial stages of dog domestication are thought to have been associated with the emergence of surplus meat available locally and produced by Late Pleistocene hunter-gatherers in Eurasia (13, 14). It has been hypothesized that when the dog ancestor entered the human commensal niche (13, 15), access to predictable food sources resulted in a reduced need for paternal care and the associated exclusivity of pair bonding (16, 17). This could have triggered the transition from the genus-typical social monogamy to polygamy (16–18). In line with this, free-ranging domestic dogs (FRDs), that largely rely on anthropogenic food, are mostly polygamous (1, 19 and see *SI Appendix* for further references). Reversal of the ecological dependence on human-derived food to hunting wildlife is rare in modern dogs and has been documented in only one western dog population in the Galápagos (20). In Australian dingoes, a shift toward hunting wild prey coincides with a predominantly monogamous mating system, with polygamous mating occurring only as an alternative strategy (21). Based on this knowledge, we hypothesize that a transition from monogamy to polygamy was one of the earlier ecologically driven adaptations to the human niche during the dog domestication process (22) rather than a trait emerging through deliberate or unintentional artificial selection by humans (23, 24).

Given that dog domestication preceded domestication of other animals (25), in its early stages humans likely lacked the means and skills to control animal breeding (22). Yet, differently from all other domesticates, dogs already showed limited introgression from wild relatives at the early stages of domestication (26), whereas gene flow from dogs into wolves was present early (27, 28) and continues to this day (26, 29). The shift to a polygamous mating system, the associated changes to canine seasonal breeding patterns and the potential effects this may have had on the formation, composition, and stability of social groups (30) may thus have facilitated the domestication process. This could be achieved by enhancing the divergence and maintenance of genetic differentiation between early dogs and their ancestors, as well as promoting the occurrence and spread of traits adaptive in the new niche of human commensal.

Characterization of the mating system of FRDs may provide insights into the canid population(s) at early stages of domestication. FRDs are a genetically distinct group rather than an admixture of breeds and may represent lineages originating from early dog populations in their respective regions, even though some FRD populations receive considerable gene flow from pure-bred dogs (31, 32). Despite a general agreement that the shift in reproductive patterns played an important role in dog evolution (16–18, 22–24), the FRD mating system has rarely been studied in detail. Male and female polygamy is the predominant mating system described in FRDs (1, 16), which was confirmed by a single genetic study so far (carried out on one FRD population), showing polygamous mating in both sexes, one case of multiple paternity within a litter and one case of father-daughter mating (17). Unlike monogamous canids, where achieving a breeder status ensures long-term access to a mate, polygamous males must develop strategies to secure access to mates. Behavioral observations suggest that both female and male mating preferences are related to social status (19, 33), and that some females may display seasonal monogamy, allowing mating by only one male during a particular estrus period (33–35). In an FRD pack living in an environment with very limited food sources in Alaska, a single female breeder was observed, implying seasonal or long-term monogyny (36). No other evidence for monogamy is present for males, and genetic analyses corroborating instances of seasonal monogamy and mate preference more generally, are lacking. Parental care is predominantly provided by the mother, although some limited care from putative fathers and alloparental care from related females has been reported in an Indian FRD population (35, 37, 38). The reported dispersal distances are small compared with the roaming capabilities of both sexes, which may be explained by negative fitness consequences of long-distance dispersal (19), although current studies are limited.

Here, we investigate the characteristics of the FRD mating system to test whether they are consistent with predictions from the hypothesis that the monogamy to polygamy transition was an ecologically driven adaptation to the anthropogenic niche during domestication. We predict that the dog mating system, while being clearly distinct from monogamy typical of gray wolves, retains some traits of this ancestral system, suggesting a natural transition between mating systems (Prediction 1). However, polygamy can intensify cross-breeding between individuals from different populations; therefore, it could have facilitated gene flow from wild wolves, hindering the domestication process. Given that introgression from wolves to dogs has been limited since the early stages of domestication (26), we predict that the dog mating system possesses characteristics that enable the reduction of gene flow from wolves (Prediction 2). Given that mating behavior plays an important role in the emergence and maintenance of novel traits in populations (39), we also predict that the dog mating system has characteristics that could have facilitated the emergence and spread of novel adaptations to the niche of human commensal in early dog populations (Prediction 3). For example, the greater flexibility of social group composition in polygamous dogs compared with monogamous wolves (17) may facilitate the integration and reproduction of dispersing individuals in new groups.

We test these predictions using whole-genome SNP array data from 526 FRDs representing three geographically distinct populations and behavioral observations from one of them. For this purpose, we analyze the kinship patterns in all three populations, focusing on identifying specific characteristics of the mating system that i) differentiate dogs from wolves, ii) contribute to the reduction of gene flow from wolves, and iii) are likely to promote the spread of novel adaptive traits in dog populations. Based on the reconstructed genealogies, we assess parameters such as the distribution of reproductive partners and number of offspring, relative numbers of maternal and paternal half-siblings, and the distribution of paternity within litters. We compare the parameter estimates from the study populations against those predicted by simulations of random indiscriminate mating. Next, we investigate factors affecting mate choice and reproductive success, which are likely to provide insights into potential mechanisms facilitating divergence from wolves. Social network data collected from one of the study populations were leveraged to obtain quantitative measures of social interactions between study individuals that may affect their mating patterns. Furthermore, we investigate social group composition and dispersal patterns, which may affect the spread of novel heritable traits as well as the rate and direction of gene flow between populations.

## Results

### Study Populations.

We reconstructed the genealogy of an FRD population from Tamraght, Morocco (*SI Appendix*, Fig. S1) for 196 individuals genotyped at genome-wide SNPs using Axiom Canine Arrays (Thermo Scientific). This genealogy was analyzed alongside genealogies reconstructed using the same methodology for two published datasets of 44 individuals from Italy (17) and 286 individuals from Ukraine (40), which were genotyped using SNP arrays: Axiom Canine Array and CanineHD BeadChip (Illumina), respectively. These populations displayed the typical characteristics of FRDs (16, 18): The dogs were not owned and could move and breed freely, but were dependent on human-derived food, obtained from direct provisioning and refuse scavenging. While all three populations were representative of FRDs, they differed in some characteristics, such as effective population size and inbreeding level (*SI Appendix*, Table S1). Therefore, interpopulation comparison allowed us to distinguish between common and population-specific characteristics of the FRD mating system.

### Network of Half-Siblings Reflects Male and Female Polygamy.

**Fig. 1.**
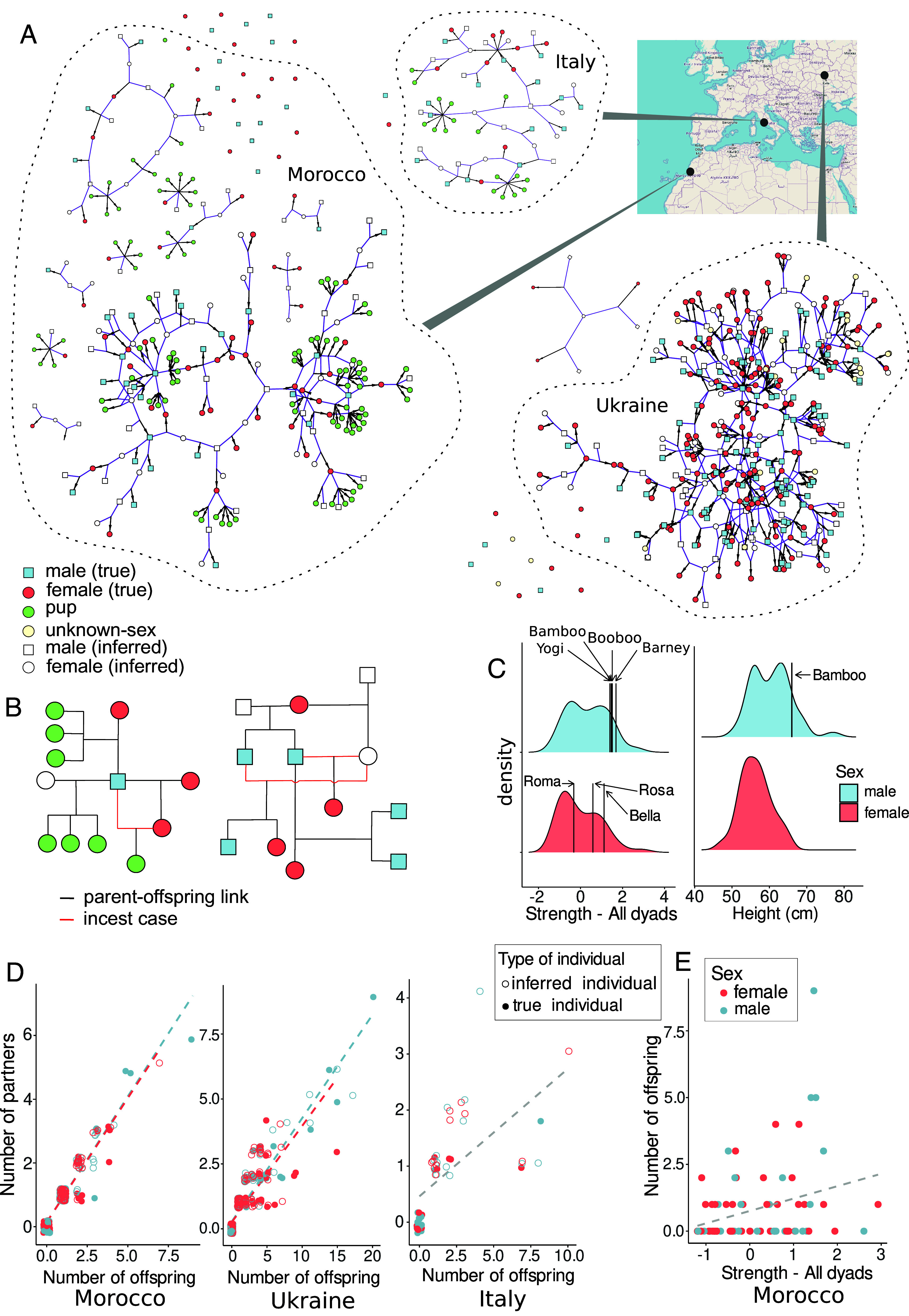
Kinship relationships in the three FRD populations studied. (*A*) Genealogies reconstructed from genetic data. The networks show relationships at the parent–offspring and sibling level, while dotted lines encircling the networks denote connections at the third and fourth kinship degree. (*B*) Examples of close inbreeding cases (marked as red lines in the family trees). (*C*) Distribution of the social network parameter “strength” values in males and females, and distribution of the height at the withers in Moroccan dogs. The values for the most successful breeders among the genotyped individuals are indicated with vertical lines. The distribution of “strength” values is based on the genotyped individuals’ values only, which were extracted from the Z-transformed data for the entire social network. (*D*) The number of inferred reproductive partners of individuals as a function of the number of offspring. (*E*) Dependence of the offspring number of individual dogs in Morocco on the social network parameter “strength”, reflecting the strength of social bonds with other individuals in the network.

We reconstructed the genealogies for the Moroccan and Ukrainian populations using multiple approaches (*Materials and Methods*), which collectively allowed us to identify first- to fourth-degree relatives with high accuracy and resolve highly complex kinship relationships. For the Italian population, we used published genealogy data (17). All three populations showed similar patterns, with a wide network of kinship relationships resulting from frequent occurrence of maternal and paternal half-siblings (Fig. 1*A* and *SI Appendix*, Fig. S2). High incidence of individuals sharing only one parent (*SI Appendix*, Table S2) reflects a mating system characterized by male and female polygamy. In each population, most individuals belonged to one main pedigree network, but each population also contained single unrelated individuals, likely representing recent immigrants. Moroccan and Ukrainian populations also included smaller pedigrees not linked by first-order kinship relationships to the main pedigree. In Morocco, they were linked to the main pedigree by more distant relationships. This was not the case in Ukraine, which suggests that some dog groups were not connected with the rest of the population by gene flow, implying that mating within the population was not random.

### Multiple Paternity within Litters Points to Female Polygamy and the Potential for Sperm Competition.

Double and triple paternity occurred in seven out of 24 litters (29%) with at least two genotyped offspring in Morocco (*SI Appendix*, Fig. S3) and in one out of four litters (25%) in Italy (in the Ukrainian population, litter membership was unknown). Frequent incidence of multiple paternity within litters points to a reduced ability of males to monopolize females. In the Moroccan population, three out of the four known fathers of multiple-paternity litters were also the top three males with the largest numbers of both reproductive partners and offspring (in the remaining cases, the fathers were inferred but not sampled, so their identity remains unknown). Single paternity litters may result either from a female mating with only one male within a single estrus, or one male fully outcompeting others in sperm competition. We cannot distinguish between these scenarios, and given the small number of multiple-paternity litters analyzed and the lack of mating observations, we cannot provide conclusive evidence for sperm competition.

### Distributions of Reproductive Partners and Offspring Numbers Suggest Limited Potential for Female Monopolization by Males.

The distribution of reproductive success, measured as the number of detected offspring, showed a moderate skew in both males and females (*SI Appendix*, Fig. S4*A*) and no sex differences (Wilcoxon rank-sum test, Morocco: W = 7,704, *P* = 0.768; Ukraine: W = 17,348, *P* = 0.8945; Italy: W = 242, *P* = 0.201). We considered offspring of any age: adults, subadults, and pups. The inclusion of pups may bias the reproductive success estimates, but the comparisons between sexes remain unbiased, because the pup mortality affects both sexes equally (i.e., the total pup mortality is the same for reproducing males and females in a population).

The percentages of adult females without offspring detected were moderately lower than males in two populations (60.7% vs. 73.8% in Morocco, 72% vs. 77.6% in Ukraine), while in Italy the difference was larger: 50% vs. 87.5% (*SI Appendix*, Table S3). Large percentage of adults without detected offspring in each population suggests limited opportunities to breed for both sexes.

The number of identified full-siblings per individual (population average varying from 1.61 to 3.29) was significantly lower than the number of maternal as well as paternal half-siblings in the Moroccan and Ukrainian populations (Kruskal–Wallis test, *P* < 10^−7^ in each population), but not in the Italian population. The number of maternal half-siblings (population average varying from 2.71 to 4.31) differed significantly from the number of paternal half-siblings (population average varying from 4.08 to 7.11) only in the Ukrainian population, while in the other two populations, the differences were nonsignificant (*SI Appendix*, Table S2). The significant overrepresentation of paternal half-siblings in the largest population may suggest that access to a larger number of females may lead to a larger variation in male reproductive success and hence a higher intensity of sexual selection in males (41). While not detectable from the number of paternal versus maternal half-siblings for the Moroccan and Italian population, the higher intensity of sexual selection in males is nevertheless suggested in all three populations from the comparison of the opportunity for selection measures (*SI Appendix*, Table S4). These measures were calculated as the squared coefficient of variation in the offspring number.

The maximum number of detected reproductive partners for females and males, respectively, was five and six in Morocco, six and seven in Ukraine, and three and four in Italy (*SI Appendix*, Fig. S4*B*), which is consistent with polygamy in both sexes. Importantly, in this study, we only detected cases of mating that resulted in reproduction, which may differ from the total number of copulations. Because reproductive partners were inferred from the occurrence of shared offspring, there was a significant positive effect of the number of offspring on the number of partners detected for each individual (*SI Appendix*, Table S5). This effect is only expected for polygamous, but not for monogamous populations. We also tested the effect of sex (in interaction with the number of offspring) on the number of partners. For the Moroccan and Ukrainian populations, we found a significant interaction between sex and the number of offspring (*SI Appendix*, Table S5). However, the effect of sex on the number of partners was small for both populations (Fig. 1*D* and *SI Appendix*, Fig. S4*C*), suggesting comparable fitness benefits of polygamous mating for both sexes. Distributions of the number of offspring and reproductive partners in males and females indicate that although males have no physiological limitation on the offspring number, they have limited abilities to monopolize females.

### Random Mating Could Not Produce the Observed Mating Patterns.

We carried out simulations of genealogies based on the demographic characteristics of the study populations (*Materials and Methods*) under random mating conditions. We compared the distribution of mating system parameters obtained from 1,000 simulated populations to values obtained from the real populations. In the purely random mating scenario, the number of full-siblings was close to zero (*SI Appendix*, Fig. S5*A* and Table S2), because in the absence of sperm competition, mating with *n* males within one estrus will likely result in a litter with *n* fathers. Therefore, we simulated a modified scenario that assumes random mating but limits the number of fathers per litter, further referred to as random mating with litter structure (*Materials and Methods*). This scenario better approximated the mating system parameters of the real FRD populations, with multiple offspring per father per litter and thus several full-siblings per individual (*SI Appendix*, Fig. S5*B*). When population subsampling was simulated for this modified scenario, the observed number of full-siblings matched the simulation predictions (*SI Appendix*, Fig. S5*C*). The outcomes of these two simulation scenarios therefore suggest that the number of potential fathers per litter may be limited either by direct male mating competition, female mate choice, and/or by the occurrence of sperm competition. We do not have sufficient observational data on the mating behavior to distinguish between these possibilities.

The simulated genealogies showed that under random mating (with and without the litter structure) maternal and paternal half-siblings are expected to occur at very similar frequencies (*SI Appendix*, Fig. S5). This result occurred despite limiting the number of offspring per female per generation in simulations, while no restrictions were imposed on the male offspring numbers. This shows that under a random mating scenario, the male reproductive success is limited by the number of available females. In all three real populations, numbers of maternal half-siblings were consistent with the predictions from the random mating simulation, which accounted for litter structure and included subsampling, while numbers of paternal half-siblings were considerably higher than predicted in the two larger populations from Morocco and Ukraine (*SI Appendix*, Fig. S5 and Table S2).

Reproductive skew, measuring the asymmetry of reproductive success between individuals of the same sex, was assessed in all three FRD populations to further elucidate observed differences between simulations and real data. In the Moroccan and Ukrainian populations, we observed a higher skewness of male reproductive success when compared with the simulation expectations, while the skewness of female reproductive success was consistent with the expectations (*SI Appendix*, Fig. S6). The Italian population did not follow this pattern, probably because of the small sample size. The male reproductive skew together with the increased frequency of paternal half-siblings relative to the simulation expectations imply a differential male reproductive success that could not be accounted for by the litter structure alone, but could be explained by mate competition and/or female choice. This pattern could not be produced by either random mating or a monogamous mating system.

### Mating Patterns Suggest Preference toward Familiar Mates.

In the Moroccan and Italian populations, where litter membership was known, we detected cases where the same parental pair produced more than one litter (*SI Appendix*, Table S6). These pairs produced between two and five litters together, suggesting the existence of long-term mate preferences, which is consistent with observational studies of the Italian population (33) and other FRD populations (19, 35). The simulations showed that the observed frequency of multiple mating between the same individuals was unlikely to occur by chance in a randomly mating population (*SI Appendix*, Fig. S5). However, long-term mate preferences do not take the form of exclusive pair bonding that is observed in wild canids (1) and do not imply monogamy. In four out of six (Morocco) and one out of four (Italy) cases of individuals that produced multiple litters with the same partner, these individuals also produced offspring with other partners. For two parent pairs that reproduced together in multiple seasons, we found evidence that one or both pair members also produced offspring with another individual within the same season (*SI Appendix*, Table S6). This pattern suggests both male and female FRDs show long-term mate preferences combined with mating with multiple partners.

### The Strength of Social Relationships Affects Reproductive Success.

For the Moroccan population, we reconstructed social networks based on the frequencies of direct interactions, which were defined as observations of pairs of individuals within 10 m proximity during behavioral observations throughout one or multiple field seasons (*SI Appendix*, Fig. S7). We considered three measures of direct social connections: “degree centrality”—the number of individuals the focal individual interacts with directly, “strength”—the sum of all direct interactions of a focal individual with any other individual (measuring the strength of all social relationships of that individual), and “strongest link”—the sum of all direct interactions of a focal individual with the individual it had the most frequent interactions with (measuring the strength of the most important relationship; see *Materials and Methods*).

We found that both measures of the strength of social relationships had a significant positive effect on the number of offspring and the number of reproductive partners (Fig. 1*E* and *SI Appendix*, Table S7). This included the opposite sex interactions and same-sex interactions for males, but not for females. The effect of these network measures remained significant when the number of offspring was corrected by considering only one offspring per parent pair per litter to measure the number of successful matings. The interaction of “strength” and “strongest link” with sex had a significant effect on the number of offspring and partners, implying that the effect of social interactions on reproductive success differed between males and females. The number of interactions (“degree centrality”) with individuals of the opposite sex had a significant positive effect on the total number of offspring (*SI Appendix*, Fig. S8 and Table S7).

We also tested the effect of extended connections based on two network measures, “eigen-centrality” (measuring the strength of both direct and indirect connections) and “betweenness” (measuring connectivity to more than one social group; see *Materials and Methods*). “eigen-centrality” is associated with social status, while “betweenness” reflects the range of social connections. Comparing the effects of these two measures allows us to assess the relative effect of social dominance and connectivity on the number of partners and reproductive success. We found no significant effects, except “eigen-centrality” for male–male connections having a marginally significant positive effect on the number of partners (GLM type II F-test, *P* = 0.049), suggesting that male dominance status affects female choice.

The distribution of “degree centrality” values in genotyped individuals differed significantly between the sexes (Fig. 1*C*), with males having higher scores on average (Wilcoxon’s rank-sum test, W = 441, *P* = 0.003). However, this difference was nonsignificant when considering all the individuals included in the social network (Wilcoxon’s rank-sum test, W = 7,770.5, *P* = 0.139). The distributions of “strength” and “strongest link” did not differ between the sexes. Males with the largest number of offspring had higher than average values of “strength” and “strongest link”, but not “degree centrality”. No trend was observed for females (*SI Appendix*, Fig. S8).

### Sexual Size Dimorphism Points to Precopulatory Sexual Selection.

The distribution of height in the Moroccan population significantly differed between the sexes (Wilcoxon’s rank-sum test, W = 2,478, *P* = 4.2 × 10^−8^). Female height did not deviate from normality (Shapiro test, N = 104, W = 0.978, *P* = 0.086), but male height did (N = 88, W = 0.962, *P* = 0.011), showing three local maxima (Fig. 1*C*). Sexual size dimorphism is typically driven by precopulatory sexual selection (42); therefore, higher than average reproductive success should be expected in larger males. We did not have sufficient data to test this, but individual males with high reproductive success were found to be larger than average in both the Italian and Moroccan populations (17, Fig. 1*C*). The sexual selection gradient was higher for males (*I* = 0.0076, N = 88) than for females (*I* = 0.0052, N = 104).

### Frequent Retention of Adult Offspring Results in the Presence of Relatives in Most Social Groups.

We used the social network approach to identify social groups in the Moroccan population based on frequencies of direct interactions between individuals (*Materials and Methods*). We found considerable intergroup variation in kinship patterns within social groups (*SI Appendix*, Table S8). The average relatedness within social groups in the Moroccan population, estimated using the PI-HAT pairwise relatedness coefficient, varied from 0.005 (implying that all genotyped individuals in this group were unrelated) to 0.205 (approaching the theoretical relatedness level for second-degree kin), with an average of 0.080. Accordingly, the percentage of individuals that were not related to any other genotyped individuals in the social group varied from 0 to 100%, with an average of 41%. The percentage of parent pairs belonging to the same social group varied from 0 to 86%, with an average of 46%. In each social group for which data were available, we identified at least one parent with a reproductive partner belonging to a different group. The percentage of adult offspring found in a natal group varied from 20 to 80%, with an average of 54%. The proportion of males and females among these adult offspring was nearly equal. This proportion, however, showed high variation among the groups and could be biased, especially in groups with the smallest sample sizes (*SI Appendix*, Table S8).

In the Ukrainian population, the average relatedness within groups (PI-HAT = 0.274) was higher than in Morocco, which is consistent with the larger number of close inbreeding cases in this population (see below). The average percentage of unrelated individuals within social groups was very low (2.4%), and the presence of such individuals was noted in only one of the three social groups. The average percentage of adult offspring found in a natal group was 94%, explaining the high relatedness within groups. The average percentage of parent pairs belonging to the same social group (51%) in Ukraine was similar to that in Morocco. Despite considerable differences between populations and among social groups, there were some consistent trends: Parent pairs originated from the same group in about half of the cases; relatives were present in most social groups as a result of frequent retention of adult offspring in natal groups.

Retention and reproduction of females in natal groups may enable cooperative breeding, which may be beneficial for females by improving pup survival rates, especially in the context of inclusive fitness (16, 19). In the Moroccan population, we found a case of cooperative pup care by three maternally related females in one social group (*SI Appendix*, Fig. S9). Their litters were born in the same month and several pups were sampled with a female other than their mother (*SI Appendix*, Fig. S3*A*). The benefits of reproducing in natal groups combined with inbreeding tolerance (see below) could have facilitated the emergence of novel phenotypic traits in early dog populations.

### Close Inbreeding Occurs with Varying Frequencies among Populations.

The Ukrainian population had higher average kinship between all genotyped parent pairs (PI-HAT = 0.145) and higher proportion of genetically related parent pairs (47% pairs with first to third kinship degree) than the Moroccan population (PI-HAT = 0.085; 15%); no data are available from the Italian population due to low number of genotyped parent pairs. This result suggests differences in inbreeding avoidance at the population level, which can result from the spatial isolation of the Ukrainian population.

In the Moroccan and Italian populations, we detected one case of breeding between close relatives (close inbreeding) each, i.e., one per 131 detected matings that resulted in reproduction (0.76%) and one per 27 detected matings (3.70%), respectively. In the Ukrainian population, we detected 17 close inbreeding cases (10.63% of all detected matings). The predominant type of close inbreeding was a father-daughter mating, which occurred in all populations. However, in the Ukrainian population other types of close inbreeding (half-siblings, grandparent-grandchild, half-uncle/aunt—half-niece/nephew) were as frequent as the cases of father-daughter mating, each being identified four times. In one instance, full-siblings reproduced together. In all cases where social group membership of both closely related parents was known, they belonged to the same group. Moreover, we found multiple cases of close inbreeding within the same families (Fig. 1*B* and *SI Appendix*, Fig. S10), as well as cases of the same individuals being involved in multiple close inbreeding cases (*SI Appendix*, Table S9). The simulations of randomly mating populations (see above) show that in the Moroccan and Italian population close inbreeding occurred at a frequency expected from random mating, while in the Ukrainian population, it occurred more frequently than expected (*SI Appendix*, Fig. S11). Given that close inbreeding occurred within social groups, it did not result from random encounters and its frequency depended on kin composition of groups.

### Kin Sharing between Distinct Spatial Locations Suggests Female-Biased Dispersal.

In the Ukrainian population, individuals were sampled in two different locations, about 15 km apart. For each individual in this population, we calculated its average genealogical distance—measured as the number of steps connecting two individuals in the genealogy—to individuals sampled in the same location vs. individuals sampled in the other location. In each case, average genealogical distances between individuals in the same location were smaller compared to distances between individuals from two different locations (*SI Appendix*, Fig. S12). However, for 26 individuals (including 20 females, four males, and two individuals of unknown sex), the average genealogical distance to the other group was lower than the average distance to own group. The sex ratio among these individuals showed significant overrepresentation of females compared with the entire sampled population, both when considering only females and males (Pearson’s chi2 = 6.022, df = 1, *P* = 0.014) and when individuals of unknown sex were also included (Pearson’s chi2 = 7.827, df = 2, *P* = 0.020). This differentiation in sex ratios suggests female-biased dispersal between the two locations, which contrasts with the similar retention rates detected for adult males and females in natal populations. This pattern may result from reduced social tolerance toward dispersing males compared to females, which may also prevent male wolves from joining FRD groups, thus reducing gene flow from wolves to dogs.

## Discussion

The genetic kinship patterns in the three FRD populations studied show that while the dogs’ mating system is distinct from monogamy prevailing in their gray wolf ancestor, it retains some ancestral traits (*SI Appendix*, Table S10), suggesting a natural, environmentally driven transition (consistent with Prediction 1 outlined in the introduction). Familiarity-driven female mate choice and inbreeding tolerance likely promotes breeding with resident animals. This, in combination with female-biased immigration to social groups and increased fertility of male dogs compared with male wolves, resulting from female polygamy (22, 43, 44), reduces the likelihood of gene flow from wolves to dogs (consistent with Prediction 2). Retention of adult offspring in social groups, together with a lack of reproductive suppression and inbreeding tolerance, facilitate the emergence and maintenance of novel adaptive traits in dog populations. Furthermore, male and female polygamy implies that individuals carrying novel adaptive traits can spread them more broadly, via increased mating opportunities for males, and in females through increased reproductive success of their male offspring. Therefore, a shift in mating system could have facilitated the spread of traits beneficial in the novel human-commensal niche in early dog populations (consistent with Prediction 3). We discuss this in more detail below.

### The Mating System in FRDs Is Polygamous but Nonrandom.

The mating system of FRD populations shows characteristics inconsistent with random mating simulations. Crucially, limiting the number of potential fathers per litter in the simulation reduced the difference between the simulated and real populations. In real populations, the number of potential fathers can be limited via pre- and/or postcopulatory selection (41, 45). Therefore, the outcome of the simulations implies the occurrence of male–male competition, female mate choice, and/or sperm competition in the study populations.

Under both simulation scenarios (with fully random mating and with litter structure), the distribution of offspring number is similar for males and females, even though both scenarios restrict the maximum offspring number only for females. This shows that under random mating, male reproductive success is limited by the number of available females, in accordance with Bateman’s principle (46). The more realistic simulation scenario, which considered litter structure and subsampling from the simulated population, provided a prediction for the numbers of maternal half-siblings consistent with those found in the real populations. However, the numbers of paternal half-siblings observed in the two larger populations were considerably higher than modeled in the simulations, which can result from high male reproductive skew (47). Indeed, these populations showed a higher male reproductive skewness (*I_δ_*) compared with the simulation expectations, which was not observed in females. Therefore, some level of sexual selection likely occurs in FRD populations, increasing the number of offspring for some males. This is further supported by higher opportunity for selection (*I*) values for males compared to females, suggesting higher intensity of sexual selection in males (48, 49). This selection could act on traits influencing male success during mate competition and/or through female choice. Moreover, the frequent occurrence of multiple paternity within litters coupled with a skewed distribution of male reproductive success suggests the potential occurrence of sperm competition. This provides a hypothesis which could be tested in the future, after more data from multiple-paternity litters are collected. Altogether, the patterns observed in the study populations show that although the FRD mating system is distinct from the prevailingly monogamous mating system of gray wolves, it is inconsistent with random mating.

Precopulatory sexual selection has been shown to correlate with male-biased sexual size dimorphism across the animal kingdom (42). In mammals, the extent of body size differentiation between males and females is closely associated with the mating system of the species, with extreme male polygamy leading to the greatest male-biased dimorphism and monogamy to moderate dimorphism (50). Therefore, sexual selection patterns may be inferred from sexual dimorphism patterns. In Moroccan FRDs, female height shows a normal distribution, which is likely shaped by natural selection, balancing between the increased success in resource competition and the increased need for nutrition with larger body size. In contrast, male height shows three local maxima. This pattern is consistent with a theoretical model for species with male and female polygamy and no paternal care. It predicts the focal phenotypic trait under natural selection to be at its optimum in females, whereas multiple equilibria between female mating preferences and natural selection can exist in males (51). This theoretical model provides an explanation for the seeming discrepancy between our conclusions on sexual selection favoring larger males and female polygamy that might weaken sexual selection.

Accordingly, we found that the sexual selection gradient is higher for males than females, implying that larger body size in males is driven by sexual selection (via female choice and/or male–male competition). Therefore, larger males should have higher than average reproductive success, which could not be tested in our study, but is supported by the available data on body height for some of the most successful males (Fig. 1*C*) (17). Observational data on FRDs show that there are large differences between males in copulatory success (33), but individual males cannot fully monopolize females (52). Therefore, even though large body size may increase reproductive success via increased success in male–male competition and/or female choice (50), smaller males may also reproduce successfully, which further explains multiple local height maxima.

### Plasticity of Canid Mating Behavior Could Have Facilitated the Natural Transition to Polygamy as an Adaptation to a New Ecological Niche.

While the domestic dog mating system characterized here is clearly distinct from the gray wolf mating system described in published studies, both systems show a considerable degree of environmental plasticity and share common traits, such as the frequent retention of offspring in natal packs (*SI Appendix*, Table S10). These findings support the first prediction from our hypothesis because environmental plasticity could have facilitated a natural transition between mating systems as an adaptive response to the ecological niche shift experienced during domestication, without the need for direct human intervention. As the new ecological niche created by humans provided predictable and accessible food (13) and paternal care was no longer critical for pup survival (1, 16), the male “sneaker” strategy, occurring with low frequency in monogamous wolf populations (5–7), could have become a predominant strategy in dogs. Moreover, reproductive suppression of adult daughters by the dominant females ceased being beneficial once both mothers and daughters could successfully raise offspring at the same time, thus contributing to each other’s inclusive fitness. Therefore, the change in reproductive patterns, often thought to be a consequence of either deliberate or unintentional artificial selection by humans (23, 24), could instead have occurred naturally as an adaptation to the new niche created by humans.

Under such a scenario, the shift in mating patterns could have occurred gradually as dog ancestors became increasingly reliant on human-derived food (11). These early domestication stages could have occurred without humans interfering with the reproduction of these canids. Therefore, the populations that were subject to the first attempts at artificial selection could have already been polygamous.

### Social Interactions Affect Dog Mating Patterns and Reproductive Success, Reducing Introgression Rate from Wolves.

We found that the strength of all direct social connections of an individual, as well as connections with individuals of the opposite sex, had a positive effect on the number of reproductive partners and the observed reproductive success. Moreover, multiple litters produced by the same parent pairs occurred with higher frequency than expected by chance, and close inbreeding occurred within social groups despite the possibility for its avoidance. This suggests a preference for familiar mating partners, in accordance with behavioral observations (33, 52). Although males gathering around the female in estrus included both familiar and unfamiliar individuals, unfamiliar males were not observed mating, received more attacks from other males and remained around the female for shorter periods (52). Behavioral observations and genetic inference thus consistently suggest the importance of social relationship strength for mate choice, even though this does not take the form of an exclusive bonding of a monogamous pair.

We found that beyond the effect of male–female associations, the strength of direct social connections between males positively influenced their reproductive success, which could be achieved through the formation of coalitions (e.g., refs. 53–55). Consistently, both direct and indirect male–male connections had a positive effect on the number of reproductive partners in males, potentially by facilitating the exclusion of unfamiliar males from mating contests (52). This mechanism may also affect dispersal patterns. In the Ukrainian population, we observed female-biased medium-range dispersal, which did not result from differential emigration rates, because we found a nearly equal sex ratio for adult offspring remaining in natal packs. Sex differences in dispersal rates depend on the relative success of philopatric and dispersing individuals in securing mates and acquiring the resources needed for reproductive success (56). In social animals, this is associated with the success of dispersing individuals in joining new social groups (57). In FRDs, dispersing females may be more successful in joining new packs than dispersing males. This could result from agonistic behavior of resident males toward unfamiliar immigrating males (52), which may be prevented from joining new groups, as they are competitors for mating opportunities. Therefore, the observed sex-biased dispersal likely results from differential immigration rates to new social groups. Dispersing males that do not succeed in joining new groups may remain loosely associated with several groups (*SI Appendix*, Fig. S7) or travel larger distances before settling.

Similar proportions of adult males and females remaining in natal packs suggest that philopatry is an equally good strategy for both sexes despite the risk of inbreeding. Philopatric males may benefit from maintaining social connections with females and other males from their natal groups, which contributes to their reproductive success via the mechanisms described above. Similarly, females may benefit from remaining in natal groups by increasing their reproductive success via alloparental care, which was found to be provided by pups’ maternal relatives in our study and earlier studies from India (37, 38). Frequent retention of adult females in natal groups may facilitate alloparental care due to inclusive fitness benefits (58). Alloparental care in wolves and other wild canids is usually provided by close relatives as well, but these are mostly older siblings of the pups (1, 16). In dogs, in contrast to wolves (1, 16), additional pup care from individuals other than the mothers may not be essential for the survival of litters, but it may still reduce pup mortality and thus increase reproductive success. The fitness benefits of kinship and familiarity within natal groups may explain high philopatry levels in both sexes despite the documented potential for long-distance roaming (19).

The female dogs’ preference for familiar mating partners provides a possible mechanism to reduce the chance of their mating with male wolves, both presently and historically. Male dogs, on the contrary, may be more likely to mate with female wolves, but this would result in dog introgression into the wolf gene pool. This is consistent with population genetic data from previous studies, showing that the wolf admixture proportions in modern free-ranging dogs are considerably lower than the dog admixture proportions in wolves across Eurasia (59), and dog introgression into wolf populations is male-biased (29). Moreover, male social coalitions that reduce access of unfamiliar males to females within stable social groups, could also contribute to reducing the chance of male wolves mating with female dogs.

In addition, enhanced male fertility in populations with female polygamy (22, 43, 44) (see also *SI Appendix*) could allow dogs to outcompete monogamous immigrant wolves. The reduced seasonality in the estrus cycle (60) resulting from the year-round availability of human-derived food enhanced female fertility (16). This would eventually lead to lack of seasonality in male dogs as well (as seasonality no longer brought fitness benefits), further increasing their reproductive advantage over male wolves. These characteristics of the dogs’ social and mating behavior collectively reduce gene flow from wolves to dogs, supporting the second prediction from our main hypothesis. Accordingly, a recent mathematical model showed that ecological niche separation in combination with mate preference jointly provide conditions required for the fast divergence of prehistoric dogs from wolves (61). Therefore, we suggest that the currently observed characteristics of FRD mating systems may have been present in the early stages of the domestication process and therefore actively contributed to that process.

### Flexible Social Group Composition Could Have Facilitated the Emergence and Spread of Traits Beneficial in the Novel Anthropogenic Niche.

Mating systems can determine the formation, composition, and stability of social groups, as well as relatedness among group members (30). A typical wolf pack, consisting of a breeding pair and their offspring from multiple seasons, is characterized by high relatedness between most of its members and a long-term bond between the breeding pair (1, 16). Availability of more predictable and accessible food resources likely reduced the need for paternal care in early dogs compared to wolves (16), which in turn limited the benefits of pair bonding for males. We found that about half of all known parent pairs in FRDs belonged to distinct but geographically proximate groups, suggesting that the mating individuals were often familiar with each other, but did not always form strong social bonds. This implies greater flexibility of the social group composition in dogs, which may enable them to adjust to human-induced changes in resource location and abundance. Groups could form ad hoc from unrelated individuals gathering around a new food source, but they could also become more stable in locations with long-term resource availability. According to theoretical predictions, reduced competition for resources combined with a lack of reproductive suppression may allow adult offspring to stay and reproduce in their natal groups (62). Indeed, we observed frequent retention of adult offspring in natal packs, which is consistent with the findings of earlier genetic (17) and behavioral studies (38, 63). At the same time, we also found some groups with low relatedness levels, which corroborates conclusions from observational studies that some dog groups may be composed of unrelated individuals (19).

This flexibility in social group composition could have facilitated the spread of novel adaptations to an anthropogenic niche (64) because i) new groups could be formed by unrelated individuals that established social affiliations by gathering around new abundant resources, and ii) unrelated females could more easily join and reproduce in new social groups, potentially introducing novel phenotypic traits. Therefore, the FRD mating system may facilitate the integration and reproduction of dispersing females, but male mate competition and female preferences for familiar mates may make it more difficult for dispersing males.

However, immigrant females may increase the reproductive success of their male offspring through multiple mating (65) and therefore facilitate the spread of novel adaptive traits between groups. Polygamous male dogs carrying novel traits have a high potential to spread them widely by mating with multiple females (which is further facilitated by year-round fertility). Even though some males may fail to reproduce, the successful males can have a large effect on the gene pool of their local population, with males carrying phenotypic traits improving fitness in the new ecological niche being more likely to achieve high reproductive success. Therefore, the transition to polygamy could have facilitated the natural spread of novel adaptive traits without human interference and improve fitness in early dog populations (66), supporting the third prediction from our main hypothesis.

In the study populations, individuals of both sexes were retained in natal groups with similar frequencies, but the overall retention rate was substantially higher in Ukraine (94%) compared to Morocco (54%). It is unlikely that these differences result from population culling, which was rare in both Ukraine and Morocco. High retention rate in natal groups in the Ukrainian population could result from its geographic isolation, which likely limited long-range dispersal. This high philopatry level coincides with the more frequent occurrence of close inbreeding in the Ukrainian population compared with the other two populations. This suggests that geographic isolation of a population combined with the capacity for demographic growth may lead to high relatedness levels within groups and frequent incidence of close inbreeding.

Mating between genetically similar individuals, which are close relatives and/or show strong genetic similarity resulting from high population-level inbreeding, increases the incidence of rare recessive traits in homozygous state, thus contributing to the emergence of new phenotypes. Therefore, retention of kin in social groups combined with inbreeding tolerance could have facilitated the emergence of novel phenotypic traits in early dog populations, given that they likely existed in isolation from each other due to their association with nomadic hunter-gatherer human groups (13). Thus, reproduction of adult offspring retained in natal groups and reduced inbreeding avoidance—characteristics that distinguish dogs from wolves (*SI Appendix*, Table S10; 67), could have facilitated the occurrence and maintenance of adaptations to the new ecological niche in early dog populations. This provides further support for our third prediction.

## Conclusions

Our results provide multiple lines of evidence supporting the predictions of the hypothesis that the monogamy to polygamy transition was an ecologically driven adaptation to the anthropogenic niche during domestication. We show that the mating systems of the domestic dog and the gray wolf, while being clearly distinct, show a large degree of plasticity, which could have facilitated the natural mating system transition as an adaptation to the new ecological niche, without human intervention. We also show that the domestic dog mating system has the characteristics that could have facilitated the domestication process by limiting gene flow into early dog populations from their wild progenitors and facilitating the emergence and spread of novel adaptive traits in early dog populations.

Our findings provide support to the hypothesis that the transition to polygamy was one of the driving forces of the dog domestication process rather than its outcome. Changes in the mating system, induced by human-driven habitat modifications, were likely an integral part of the domestication process. It is likely that the first attempts at artificial selection were applied to polygamous protodog populations rather than monogamous wolves. Therefore, studies on the effect of the mating system on the physiology and behavior of FRDs may provide important insights into the population(s) at early stages of domestication.

## Materials and Methods

### Study Populations, Sample Collection, and Genotyping.

We collected DNA samples from an FRD population in Morocco, from a region encompassing 6 km of beach and neighboring villages between Taghazout and Aourir (*SI Appendix*, Fig. S1). Individuals in this population form relatively stable groups around food sources. All FRDs included in the study were individually recognized based on their morphological traits, coat color, sex, and age class. We collected 208 saliva samples from 202 individuals using noninvasive collection kits (Performagene PG 100, DNA Genotek, Canada) and extracted DNA according to the manufacturer’s instructions. The samples were genotyped in two batches, the first using Axiom Canine Genotyping Array A and the second using Axiom Canine HD Genotyping Array (Thermo Scientific). The batches were merged and SNPs common to both batches were retained.

The results obtained for the Moroccan population were compared with results from previously studied populations from the suburbs of Rome, Italy (17), and Chornobyl, Ukraine (40). The Italian population lived at a nature reserve characterized by open grassland interspersed with woodland, and at the time of sampling consisted of 97 adult or subadult individuals (17). The Ukrainian population occupied areas directly adjacent to the Chornobyl Nuclear Power Plant as well as the 15 km distant Chornobyl city and was estimated at over 800 individuals (40). Unlike the Moroccan and Italian populations, the Ukrainian population was isolated by extensive woodlands, uninhabited by humans. Both the Moroccan and Italian populations showed genetic similarity to other free-ranging dog populations and were not a mixture of dog breeds, although some residual gene flow from breeds was possible (12, 17). The Chornobyl population originated from dogs abandoned after the nuclear disaster and its gene pool was a mixture of local village dogs and a wide range of breeds (40). Samples from the Italian and Ukrainian populations were genotyped using the Axiom Canine Genotyping Array (Thermo Scientific) (17) and the CanineHD BeadChip (Illumina) (40), respectively.

For the Moroccan and Italian populations, the sex and age group (pup/juvenile, subadult, adult) of individuals was known, and pups were usually found next to their presumed mother. In the Ukrainian population, the age of individuals was unknown, and sex was inferred from chromosome X heterozygosity; in several individuals, this inference was ambiguous, and therefore, their sex remained unknown (40).

### Data Filtering and Pairwise Relatedness Analysis.

For the Moroccan population, we used a dataset of 163,594 autosomal SNPs for 196 individuals, after removing 6 individuals with more than 10% missing data (for details, see *SI Appendix*). For the Ukrainian population, we used the published dataset from Spatola et al. (40), containing 286 individuals (we excluded individuals from the 45-km distant town of Slavutych) and 129,497 autosomal SNPs. The SNP data from the Moroccan and Ukrainian populations were analyzed using the same workflow (see below). The Italian dataset (44 individuals) was analyzed previously using similar methods (17), and we therefore used published results for the comparison with those from Morocco and Ukraine.

We used Plink1.9 software (68) for data filtering (*SI Appendix*, Table S11) and to estimate relatedness using the pair-wise identity by descent (IBD) coefficient PI-HAT, as well as heterozygosity, *F_IS_* coefficient, and within-individual inbreeding coefficient. We also obtained KING pairwise relatedness estimates (69) using Plink2.0.

### Reconstruction of Kinship Relationships and Genealogies.

We reconstructed genealogies using several approaches implemented in COLONY (70), CERVUS (71), and PRIMUS (72). Together, these software allowed us to resolve highly complex pedigrees and identify first- to fourth-degree relatives with high accuracy. PRIMUS analysis was based on point estimates, i.e., PI-HAT coefficients calculated in Plink based on the full set of loci (after the exclusion of loci that were nonvariable or had low genotyping rate). In contrast, COLONY and CERVUS assessed allele matching patterns between parent–offspring pairs/trios and sibling groups for each locus separately. This is computationally intensive and required a reduction of the number of loci used (*SI Appendix*, Table S11). We used COLONY as the main source of genealogical information and complemented it with the results from the two other software. In case of discordance between the three software, we used the PI-HAT and KING coefficients as well as the allele sharing patterns between pairs of individuals to identify their most likely kinship relationship. All the analyses were carried out without providing any information about individuals except for their genotypes and sex. For the Moroccan population, we tested the results against known age and mother–offspring relationships, all of which were correct except cases of pup swapping between females (*SI Appendix*, Fig. S9).

The consensus between the results from different software was determined for parent–offspring, full-sibling and half-sibling relationships. Other types of second-degree kinship (grandparent-grandchild, uncle/aunt-nice/nephew) as well as third-degree kinship (first cousins, great-grandparents, great aunt/uncle), which were not obtained indirectly from the first-degree kinship, were inferred only in PRIMUS. These last results were used to check whether individuals that did not have parents, offspring, and siblings in the sampled population had more distant relatives there. This allowed us to identify, by exclusion, individuals that were not related to the rest of the population. For details, see *SI Appendix*.

The final genealogy was drawn in R v.4.4.1 (73) as a network with the R package igraph v.2.0.3 (74). To display full-sibling relationships, nodes were manually created by connecting all the offspring edges from the same parent pair node. Next, we used the second- and third-degree kinship relationships inferred in PRIMUS to identify links between the distinct pedigrees established by parent–offspring and sibship analyses. Because PRIMUS identified multiple links between the pedigrees at these kinship levels, their clear graphical presentation was impossible. Therefore, we show pedigree groups linked by kinship relationships, but without showing specific relationships (Fig. 1*A*).

### Reproductive Success, Polygamy, and Other Individual Trait Estimates.

Based on the genealogies reconstructed for the three populations, for each adult individual we calculated i) the number of offspring present in the dataset, ii) the number of different reproductive partners an individual produced offspring with, iii) the number of full-siblings, maternal half-siblings, and paternal half-siblings present in the dataset, and iv) the number of different litters from the same parent pair. In cases where only one of the parents of an individual was present in the dataset, COLONY inferred the second (nonsampled) parent, and deduced its partial genotype based on the genotype of the first parent and the offspring. If several individuals shared the same nonsampled parent, a complete genotype of this parent could be inferred. This way, gaps in the genealogy resulting from the nonsampled relatives were filled.

Because for each individual with only one sampled parent the second parent was inferred, the number of offspring present in each dataset was the same for both sexes when considering jointly sampled and inferred parents (*SI Appendix*, Table S4). For these joint sets of parents, we estimated sexual selection gradients for males and females, based on the offspring number as an estimator of fitness. For this purpose, we calculated the Crow’s index (*I*), i.e., the squared coefficient of variation in offspring number (48) for males and females. In addition, we calculated the Morisita *I_δ_* index of reproductive skew (75), which was identified as the best approach to represent the proportion of dominant males independent of sample size (76). We calculated it for all males and all females, as well as for the subset of individuals that were parents of sampled litters (i.e., where multiple individuals from the same litter were genotyped). The *I_δ_* values from the real populations were compared with the distribution of values obtained from the simulations (see below).

In datasets from Morocco and Italy, besides calculating the total number of offspring for each individual, we also calculated a reduced offspring number by counting only one pup per litter per parent pair. This way we corrected for any potential bias introduced by nonrandom sampling of pups from a litter, which were frequently sampled together. This also provided a measure of the number of successful matings per individual (i.e., matings resulting in offspring).

We calculated the number of all offspring (of any age) identified for each individual. The proportion of pups among all the offspring considered was 47.4% in Morocco and 42.2% in Italy, while no pups were sampled in Ukraine. Including pups may bias the reproductive success assessment because of high pup mortality. However, we calculated the reproductive success in order to compare it between males and females, and collectively females lose the same number of pups as males. Therefore, the comparisons between sexes remain unbiased. The calculation of the reduced offspring number mentioned above allowed us to further reduce this bias source.

Reproductive partners were identified based on shared offspring; therefore, a positive correlation between the two variables may be expected in polygamous but not in monogamous populations (where the number of offspring may grow without any increase in the number of partners). The potential effect of the number of offspring on the number of identified reproductive partners was tested by constructing a general linear model with Poisson distribution using the MASS package v.7.3-61 (77) and applying a type II F test from the car package v.3.7.6 (78) on the predictors, which were the number of offspring, sex, and its interaction with the number of offspring. In addition, the potential effect of sex on the number of partners was compared with random expectations by visual comparison of the curves obtained from the cumulative distribution function applied to empirical data on the number of partners for males, females, and all individuals together with the curves obtained from 1,000 permutations. The number of full siblings present in the dataset was compared with the number of maternal and paternal half-siblings using a nonparametric Kruskal–Wallis test, and post hoc multiple comparisons. Lack of significant differences would imply similar distributions of reproductive success in both sexes, while the significant overrepresentation of paternal half-siblings may suggest a larger variation in male reproductive success (47), resulting in a higher intensity of sexual selection in males (41).

### Analysis of Kin Structure in Social Groups.

We identified social groups in the Moroccan population based on direct observations and a social network analysis (see below). We used the hierarchical clustering algorithm—*cluster_fast_greedy*—implemented in R-package igraph (74) to identify groups of individuals based on pairwise proximity within 10 m distance, calculated separately for each field season (2016-17, 2017-18, 2018-19, and 2019-20). In addition, we plotted the social network structure using the Fruchterman–Reingold layout algorithm implemented in igraph for the same data and visually delimited social clusters in these plots (*SI Appendix*, Fig. S7). Based on the results from both methods (which showed good consistency), we assigned each individual to the cluster it had the most and strongest connections to, and in the case of ambiguities left individuals unassigned. Finally, we compared these results with the observations of individuals being observed with their assigned group in the field. For some groups (named K17, K18, Groom; *SI Appendix*, Fig. S7), we did not have the pairwise proximity data from all the field seasons. However, these groups were spatially separated from other groups studied, so the membership of these groups could be assigned with high certainty based on the existing data.

In Morocco, we analyzed kin structure in 11 groups that included at least three genotyped individuals (*SI Appendix*, Table S8). In the Ukrainian population, we identified three social groups based on their sampling in isolated locations where dogs were regularly fed (40). The remaining individuals had unknown group affiliations and were excluded from this analysis.

We assessed the average relatedness within social groups by calculating the average pairwise PI-HAT and KING coefficients across all pairs of genotyped individuals within each group. We also identified individuals that were not related to any other individual from their group and calculated the proportion of such individuals within groups. In this analysis, we considered a pair of individuals as unrelated if their relationship identified in PRIMUS was either “unrelated” or “distant kin” (which corresponds to the kinship of the fourth degree and below). We combined these two kin categories, because PRIMUS frequently indicated uncertainty in distinguishing between them. We also calculated the proportion of parent pairs from the same group among all parent pairs with known group affiliation, and the proportion of adult offspring found in a natal group (i.e., their mother’s group) among all adult offspring with known group affiliation.

### Multiple Paternity and Litter Sharing.

In the Moroccan dataset, we checked whether mothers assigned to individuals sampled as pups were consistent with prior information on maternity of litters based on behavioral observations. A mismatch between the expected mother and the mother inferred from the parentage analysis may result from sample mislabeling (which was checked in each case) or—if it involved two mothers for pups from the same litter—a potential litter sharing (alloparental care). In the second case, spatial proximity of dens of both mothers and temporal proximity of both litters’ births was checked. In addition, we also identified fathers of each litter. Cases of multiple paternity were identified as more than one father assigned for different pups from the same litter.

### Identification of Close Inbreeding Cases.

We assessed the relatedness levels inferred for parent pairs in a pedigree to identify possible cases of close inbreeding, i.e., mating between close relatives. The cases considered ranged from the first to fourth kinship degree: parent–offspring and full siblings (first degree); half siblings, grandparent–grandchild and aunt/uncle–niece/nephew (second degree), half-aunt/half-uncle–half-niece/half-nephew, full first cousins, great-grandparent–great-grandchild (third degree), and half first cousins (fourth degree). In the presence of multiple types of inbreeding for the same parent pair (e.g., if they are themselves the result of mating between close relatives), only the higher degree of kinship was considered. We compared cases of close inbreeding observed in the three empirical datasets with those obtained from random mating simulations.

### Simulations of Randomly Mating Populations.

We carried out pedigree simulations using the R package MoBPS (79) for each study population. All simulations involved a founder population, followed by successive rounds of mating up to the number of concurrent generations seen in the real populations. Two sets of simulations under different mating scenarios were done for each population: a fully promiscuous (i.e., random) mating system, and a second one matching the known litter structure of dogs, i.e., the distribution of litter sizes and the number of fathers per litter. For both simulations, reproductive constraints were set for females to reflect physiological limits to the production of offspring per generation (*SI Appendix*, Fig. S13 and Table S12), but no limit was assumed for males. This design resulted in six simulation scenarios, each with 1,000 independent simulations. From each simulated genealogy, we randomly sampled the number of individuals corresponding to the number that was sampled and genotyped in each empirical population (*SI Appendix*, Table S1). For details, see *SI Appendix*.

For each simulation scenario, we assessed the distribution of i) the number and type of close inbreeding cases, ii) average number of different litters by the same parent-pair (i.e., litters with the same parents in different simulation generations), and iii) average number of full siblings, maternal half-siblings, and paternal half-siblings. We compared the distributions of these parameters within the simulated datasets with empirical data from each population. This was done for both complete genealogies and those randomly subsampled, as described above. Male and female reproductive skewness (*I_δ_*) were calculated on all simulated datasets as described in ref. 75, using the number of offspring as the measure of reproductive output, and compared with observed values for the three populations. Two different datasets were used: the complete dataset including all offspring, and a dataset comprising only offspring identified as part of litters (i.e., offspring sampled as pups in the Moroccan and Italian populations, and offspring with at least one maternal half-sibling or full-sibling for the simulated populations).

### Effect of Social Network Measures on Reproductive Success.

Individual mating patterns and reproductive success may be determined by social interactions among individuals and the structure of social groups. The social network approaches can be used to assess the intensity of both direct and indirect social connections and provide quantitative measures of individual social interactions, which makes them particularly relevant to study mate choice, mating success, and sexual selection (80).

We used social network measures to assess potential links between the way an individual interacts with conspecifics and its reproductive success. We observed social interactions in Moroccan dogs along transects of predetermined scan areas during five field seasons between 2016 and 2024 (for details, see *SI Appendix*). Social network measures were calculated based on the number of observations of pairs of individuals within 10 m distance. Such a single observation was considered as a “direct interaction.” We calculated the number of such interactions across all individuals as well as for male–male, male–female, and female–female dyads separately.

Five network measures were considered as potential predictors of the number of reproductive partners and the number of offspring: i) “degree centrality” (the number of individuals a focal individual had direct interactions with), ii) “strength” (the sum of all direct interactions of a focal individual with any other individual), iii) the“strongest link” (the sum of all direct interactions of a focal individual with the individual it had the most frequent interactions with), iv) “eigen-centrality” (the sum of “strengths” of a focal individual and all individuals it had a direct or indirect interactions with; a high “eigen-centrality” score means that an individual is connected to many individuals who themselves have many connections), and v) “betweenness” (the number of times an individual is situated on the shortest path between other individuals—a measure that achieves high values for individuals connected to multiple social groups).

To test the effect of these network measures on the number of offspring and the number of reproductive partners per individual, generalized linear models with negative binomial distribution were constructed and the effect of predictors was tested using type II F tests. The predictors used were the social network measure, sex, and the interaction of the network measure with sex. Sex did not have a significant effect on the number of offspring, but its interaction with some network measures significantly affected the number of offspring. For the models where the interaction with sex was significant, we additionally ran type III F tests.

We considered the proportion of days an individual was observed during the different sampling periods as a potential confounding variable, because it may influence the social network and reproductive success measures. We carried out the analyses both without and with the inclusion of this confounding variable and found that most results remained unaffected (*SI Appendix*, Table S7). We also tested for differences in the distribution of the network measures between males and females (for the genotyped individuals only and for all the individuals), using Wilcoxon’s rank-sum test. For details, see *SI Appendix*.

### Analysis of Sexual Size Dimorphism.

In the Moroccan population, we measured the height (at the withers) for 237 individuals. The measurements were taken either directly or from videos of an experiment in which the subject stood close to a known experimenter. The dog height was calculated in relation to the known leg length of the experimenter. The mean error of the indirect measurement was 3.05 cm, and both measurements were highly correlated (N = 21, R = 0.82, *P* < 0.001). Due to the low overlap between the individuals for which the height was known and for which the genetic data were available (N = 15), these height data were not compared with reproductive success, but their distribution in males and females was analyzed. We calculated sexual selection gradients for body height in males and females, using the same method as in the case of gradients for offspring numbers. We also tested for differences in the distribution of height between males and females using Wilcoxon’s rank-sum test and assessed the normality of the sex-specific distributions using the Shapiro test.

## Supplementary Material

Appendix 01 (PDF)

Dataset S01 (XLSX)

Dataset S02 (XLSX)

Dataset S03 (TXT)

## Data Availability

Datasets S1–S3 containing the reconstructed genealogies and offspring numbers, social network data, and R scripts used for the data analysis as well as additional files containing SNP genotypes have been deposited in Figshare repository (DOI: 10.6084/m9.figshare.27323778). Previously published data were used for this work (17, 40).
