# Supplementary material for: Mating system of free-ranging domestic dogs and its consequences for dog evolution
Source: Proc Natl Acad Sci U S A. 2025 Nov 24;122(48):e2421756122. doi: 10.1073/pnas.2421756122 (PMC12684915; doi:10.1073/pnas.2421756122)
Supplement: Supplementary file 1 — Appendix 01 (PDF) [file pnas.2421756122.sapp.pdf]

Supporting Information for
Mating system of free-ranging domestic dogs and its consequences for dog evolution

Clément Car[a], Roya Adavoudi[a], Andreas Berghänel[b,c], Melissa Vanderheyden[b], Andre E. Moura[a], Friederike Range[b], Giulia Cimarelli[b,d], Martina Lazzaroni[b,e], Rachel Dale[f], Ikhlass El Berbri[g], Gabriella J. Spatola[h,i], Timothy A. Mousseau[i], Sarah Marshall-Pescini[b]*, Małgorzata Pilot[a]*

a Department of Evolutionary Genetics and Biosystematics, Faculty of Biology, University of Gdańsk, 80-308 Gdańsk, Poland
b Domestication Lab, Konrad Lorenz Institute of Ethology, Department of Interdisciplinary Life Sciences, University of Veterinary Medicine Vienna, 1160 Vienna, Austria
c Zoology and Animal Ecology Research Group, Department of Biology, University of Hildesheim, Hildesheim 31141, Germany
d Behavioural Ecology Group, Wageningen University and Research, 6708 WD Wageningen, Netherlands
e Department of Chemistry, Life Science and Environmental Sustainability, University of Parma, 43124 Parma, Italy
f Department for Psychosomatic Medicine and Psychotherapy, University for Continuing Education Krems, 3500 Krems, Austria
g Department of Veterinary Pathology and Public Health, Agronomy and Veterinary Institute Hassan II, 10101 Rabat, Morocco
h Cancer Genetics and Comparative Genomics Branch, National Human Genome Research Institute, National Institutes of Health, Bethesda, MD 20892, USA
i Department of Biological Sciences, University of South Carolina, Columbia, SC 29208, USA

*corresponding authors: sarah.marshall@vetmeduni.ac.at, malgorzata.pilot@ug.edu.pl

This PDF file includes:

SI Methods
SI Additional information based on the literature
Figures S1 to S13
Tables S1 to S12

Other supporting materials for this manuscript include the following:

Additional resource – SNP genotypes from the Moroccan dogs used to reconstruct the genealogy
Dataset S1 – Reconstructed pedigrees including all the individuals sampled in the three populations
Dataset S2 – Social network data for the Moroccan population
Dataset S3– R scripts used for the data analysis
These datasets have been deposited in Figshare repository:
doi.org/10.6084/m9.figshare.27323778

Supporting Information Text

Extended Methods

Genotyping arrays used to obtain the three FRD datasets studied. The saliva samples collected non-invasively from the Moroccan dogs were genotyped in two batches, the first using Axiom Canine Genotyping Array A and the second using Axiom Canine HD Genotyping Array (Thermo Scientific). The first batch was collected and genotyped before the HD array was available. The two batches were merged and SNPs common to both batches were retained.

The data from the two other populations were obtained from earlier studies. In the Italian population, samples were obtained from tissue remaining after sterilisation and genotyped using the Canine Axiom Genotyping Array A (Thermo Scientific) (1), while in the Ukrainian population blood samples were obtained and genotyped using the CanineHD BeadChip (Illumina) (2). The datasets are available from the following repositories: Morocco - Figshare (doi.org/10.6084/m9.figshare.27323778 ; this study), Ukraine - GEO (accession ID GSE219090; 2), and Italy – Dryad, doi: 10.5061/dryad.stqjq2c2q (3).

Sterilisation in the study populations. Each study population was subject to sterilisation as a population control method. In the Italian population, 25% of individuals were sterilized (1). In the Ukrainian population, the genetic samples were collected at the time of sterilisation (2) and therefore they reflect the mating patterns before sterilisation, but the number of sterilised individuals grew as the sample collection progressed. In Morocco, dogs were occasionally sterilised by animal welfare organisations and their frequency was estimated at 13%. Sterilisation is common in modern FRD populations, but most likely did not occur in early dogs. The genetic effect of sterilisation corresponds to that of the animal's death, and at moderate frequencies is unlikely to change the mating system in the affected population.

Data filtering. We used Plink1.9 software (4) to remove SNPs with a genotyping rate below 90% and minor allele frequencies below 1%, and to carry out further filtering steps listed in Table S11. For the Moroccan population, the merging of the two genotyping batches resulted in a dataset of 163,594 autosomal SNPs for 208 samples. We then removed from this dataset six individuals sampled twice (after their identity was confirmed by matching genotypes) and six individuals with more than 10% missing data. Exceptionally, we kept one genotype of a pup with 15% missing data, because it was correctly assigned to its known mother and being a pup could not cause any other genealogy errors (i.e. could not be a parent). The final dataset consisted of 196 individuals.

In the next step of loci filtering applied to both the Moroccan and Ukrainian population, we removed loci in strong linkage disequilibrium (with $r^2>0.1$ within 50 consecutive SNPs, shifted and recalculated every 10 SNPs). We then applied filters to retain only the loci with the highest heterozygosity for the COLONY and CERVUS analyses, where the full SNP set could not be used due to computational limitations (see Table S11). We did not apply identical filters for each population, because the filtering was adjusted to obtain high-quality SNP datasets of required sizes that could be used for the kinship analyses.

Pairwise relatedness analysis. For each population, we used Plink1.9 to estimate relatedness using the pairwise identity by descent (IBD) coefficient PI_HAT, as well as population genetic parameters such as heterozygosity, F_{IS} coefficient and within-individual inbreeding coefficient. We also used Plink2.0 to estimate relatedness using KING pairwise relatedness estimates (5). These calculations were done for the datasets after the first filtering step (retaining SNPs with no more than 10% of missing data and MAF<1%; Table S11).

Details on genealogy reconstruction. We reconstructed genealogies using several approaches implemented in COLONY (6), CERVUS (7) and PRIMUS (8). We used COLONY as the main source of genealogical information, because of several characteristics that were particularly useful for the analyses of incomplete datasets (i.e. dataset that did not include all individuals from the study populations). COLONY identifies parent-offspring pairs and trios as well as full and half-siblings among sampled individuals, but also infers unsampled parents. Therefore, it can identify

a shared parent of half-sibling groups, this way filling the gaps resulting from incomplete sampling. Because COLONY identifies kinship relationships based on allele-sharing patterns in individual loci, it can accurately distinguish between parent-offspring and sibling pairs. Moreover, the software can establish the direction of parent–offspring relationships without age information by considering all the inferred kinship relationships of each individual (e.g. there may be multiple siblings with the same mother, but not with the same offspring). We carried out the COLONY analysis without providing any information about individuals except for their genotypes and sex. We then tested the results against known age and known mother-offspring relationships, all of which were correct except cases of pup swapping between females within a social group (Figure S9).

The COLONY analysis was done using the full-likelihood method. The offspring list included all sampled individuals, which allowed the detection of parents of adult individuals as well as pups. The lists of potential mothers and fathers included only adult individuals (except the Ukrainian population where individual age was unknown, hence all individuals were considered as potential parents). We allowed the option of polygamous mating for both males and females, which does not exclude the possibility of monogamy. We also allowed the occurrence of inbreeding, but the software failed to detect some cases of father-daughter breeding (detected based on the total evidence from the other methods). Therefore, we repeated the runs with the same parameters, but listing these cases as known parent-offspring relationships (see Table S9).

Another analysis based on allele sharing patterns was performed using CERVUS software. Although CERVUS is designed to infer parentage, it can also be used to identify sibling pairs. These are inferred as putative parent-offspring pairs, but with too large numbers of mismatching loci (i.e. inconsistencies in the expected parent-offspring allele sharing pattern) compared with true parent-offspring pairs. The confidence levels for the inferred parent pairs were established by simulating populations with the same allele frequencies as the study population. We carried out simulations of parent-offspring pairs and trios for 10,000 offspring produced by the same number of parents as in the study population, and applied a strict confidence level (95%). In practice, via comparisons with COLONY and PRIMUS outputs, we found out that the small number of mismatching loci (<10) is a better indicator of the true parent-offspring relationship than the confidence level alone. Most pairs identified in CERVUS as parent-offspring with 95% confidence, but with larger number of mismatches were identified as siblings in COLONY and PRIMUS.

Pairwise kinship relationships were also inferred using the PRIMUS software. PRIMUS uses pairwise PI-HAT coefficients obtained in Plink to infer kinship relationships of first-, second- and third degree, and reconstruct the most likely pedigree. We ran PRIMUS with default parameters, except for the maximum gap between individuals that produce offspring, which was set to 3 generations. PRIMUS inferences of parent-offspring pairs, full-siblings and half-siblings were compared with those obtained in COLONY and CERVUS. Other types of second-degree kinship (grandparents-grandchildren, aunt/uncle-niece/nephew) as well as third-degree kinship (first cousins, great-grandparents, great aunt/uncle) were inferred directly only in PRIMUS. Therefore, we used these more distant kinship inferences to identify relatedness links between distinct pedigrees established by parent-offspring and sibship analyses. We also tested whether individuals that did not have parents, offspring and siblings in the sampled population had other more distant relatives there.

The parentage analysis was carried out in two steps. First, we carried out the analyses in COLONY and CERVUS without providing any information about the study individuals except their sex and potential to participate in reproduction (pups and individuals with unknown sex were excluded from the list of potential parents). We then cross-checked the results of both analyses. Each parent-offspring relationship between genotyped individuals identified in COLONY was compared with the CERVUS parentage assignment. In the reverse direction, each parent-offspring relationship identified in CERVUS and with less than 10 mismatching loci was compared with COLONY results. Next, both parent-offspring and sibling relationships inferred in COLONY were compared with the PRIMUS results. In the case of discordance between the three software, we used the PI-HAT coefficient and the genome-wide allele sharing patterns between two individuals (Z coefficients) obtained in Plink to identify their most likely kinship relationship. For the Moroccan population,

information about the age of individuals was also used to assess the reliability of the inferred direction in parent-offspring relationships.

From these comparisons, a temporary consensus parentage reconstruction was generated. The inference was highly consistent between the three software and the relatedness coefficients. Inconsistent inference occurred in cases of mating between close relatives, in particular between fathers and daughters, which were incorrectly inferred in both COLONY and PRIMUS, but detected based on CERVUS results and genome-wide allele sharing patterns (Z coefficients).

In the case of the Ukrainian population, an additional CERVUS run to detect parent-offspring trios was performed, which considered all individuals, including those without sex information, as potential parents, and assumed that sex of all individuals is unknown. This allowed us to identify four individuals without sex information as mothers, based on the known sex of the second parent in the trios.

A second COLONY run was then conducted for each location, by introducing the inferred father-daughter close inbreeding cases as known relationships in the input file, and including new information about the sex of four dogs from Ukraine. All the parent-offspring relationships inferred from the second run of the COLONY software were finally compared with CERVUS and PRIMUS results, PI_HAT, KING and Z coefficients. Next, we used the results from PRIMUS for the second- and third-degree kinship relationships to identify links between the distinct pedigrees established by parent-offspring and sibship analyses. This allowed us to identify, by exclusion, individuals that were not related to the rest of the population.

Simulations of randomly mating populations. Pedigree simulations under two different mating scenarios were carried out using the R package MoBPS (9). Independent simulations were carried out for each study population (Morocco, Italy and Ukraine), to match their census population sizes and the number of concurrent generations present (Table S1). All simulations were done by first creating a founder population using the function creating.diploid, with each successive generation produced using the function breeding.diploid. For each breeding.diploid step, all males and females from all previous steps were used as potential reproductive partners. The breeding.diploid step was then repeated as many times as needed to create the same number of concurrent generations as in the real population. The number of individuals produced in each step was calculated by dividing the census population size by the number of concurrent generations, and rounding it up to the natural number. Sex ratio was always set to 0.5.

For each population, two sets of simulations were carried out: one simulating a fully promiscuous (i.e. random) mating system, where each individual has the same probability of mating with any other individual of the opposite sex, and each mating was equally likely to produce offspring; and a second one matching the litter structure of dogs, i.e. with limited number of fathers per litter. For the random mating simulations, the only constraint was set for the maximum number of offspring per female at 11 in each generation (although each offspring could be from a different male). This reflects the reproductive constraints for females known from the study population in Morocco. No limit was assumed for males.

For the litter structure simulations, each mating pair produced multiple offspring per generation using the option repeat.mating, set to a probability distribution for litter sizes from one to eight. This probability distribution was determined based on field data for litter size estimated from Morocco, but considering only the litter sizes up to eight (Table S12). This limitation was necessary to limit the maximum number of offspring per female per generation to realistic values (see below). For each generation, the maximum number of matings per pair was then set to 10, with no further limitations for males. The simulations only reflect the matings that resulted in the production of pups. For females, the number of different partners per generation was limited to three, to reflect the fact that we never observed litters with more than three fathers in the study populations. Therefore, the maximum number of offspring a female could produce each generation was 24, although in the resulting simulations this occurred rarely. In most cases, the resulting pedigrees had no more than 15-17 offspring per female per generation (Figure S13), which was closer to the litter numbers observed in the Moroccan population (Table S12).

This design resulted in six simulation scenarios in total, each with 1000 independent simulations with random seed numbers, and with the final pedigree being produced for each generation present in the simulations using the get.pedigree function. From each reconstructed genealogy, we randomly sampled the number of individuals corresponding to the number that was sampled and genotyped in each empirical population (Table S1), using the *sample* function in R. This allowed us to assess the effect of incomplete population sampling on the kinship inference.

Social proximity data collection. We recorded social proximity observations in Moroccan dogs during five field seasons between 2016 and 2024, applying proximity scans along transects of predetermined scan areas (Figure S1). For different scan transects, the order of the scan areas during the transect was randomly assigned to minimize systematic interdependence effects between adjacent and consecutive scans. During the first three seasons (season 1: September 2016 – January 2017 [43 scans], season 2: October 2017 – February 2018 [40 scans] and season 3: October 2018 – March 2019 [65 scans]), scans were conducted every three days, recording pairs of individuals seen within 10m distance. From October 2019 we started continuous observation, which was carried out in season 4 (until March 2020) and season 5 (April 2022 – September 2024), with an interruption due to Covid19 lockdown. During these two seasons, scans were conducted on an almost daily basis, recording the precise GPS location of each individual on a handheld tablet using Map Marker, which allowed us to calculate dyadic distances on a continuous scale up to 50 m distance (season 4: 94 scans, season 5: 819 scans). We recorded all associations within 50 m for each individual in the scan area, including with individuals outside the scan area if they were within this range.

Effect of social network measures on reproductive success. We used the social proximity data on the Moroccan dogs, collected during seasons 1-4 (for which the genetic data were available) to reconstruct social networks, using the R-package igraph. Social network measures were calculated based on counts of observations of a dyad within 10 m distance, which was considered as a “direct interaction”. We followed the recommendations of Hoppit & Farine (10) to account for the sources of error that may affect the network measures, such as mortality and dispersal of individuals during the observation period, missed observations of dyadic interactions outside the scan area as well as differences in sampling frequency over time. Therefore, the counts of “direct interactions” for each dyad were calculated considering only the period of observations from the first day to the last day both individuals were observed during the scans. For that period, we calculated the number of times the dyad was seen within the distance of 10 m, divided by the number of times when at least one of these individuals was seen.

Due to the very high and wide visibility in our study area, individual recognition error (i.e., the likelihood of missing a record of two individuals being associated during a scan; 10) is very low and ignored for our analyses. Occasionally, the same individual was seen multiple times in different scan areas during the same scan trajectory. We included these events because they reflect true interactions. However, we excluded all observations where an individual was observed repeatedly during adjacent and consecutive scans, as this individual may simply have followed the observer. We excluded individuals that were seen less than five times altogether during field seasons 1-3 with comparably few scans (40-65 scans per season) and less than 10 times for season 4 (94 scans). We considered the connections across all individuals as well as for male-male, male-female and female-female dyads separately.

Five network measures were considered as potential predictors of the number of reproductive partners and the number of offspring: (i) *degree centrality*, (ii) *strength*, (iii) *the strongest link*, (iv) *eigen-centrality* and (v) *betweenness* (for definitions see main text). *Degree centrality* and *strength* are the measures of direct social connections of an individual, which may affect male access to females and female mate choice. To further assess the role of social relationship strength, we also considered the strength of only the strongest relationship of an individual (*strongest link*). *Eigen-centrality* and *betweenness* are the measures of indirect connections, which may be important for male reproductive success. *Eigen-centrality* measures the strength of both direct and indirect connections, which may reflect the supportive power a male can rely on in polyadic conflicts during mating competition. *Betweenness* measures how often an individual lies on the shortest path between all dyads in a network. Its highest values are assigned to individuals

that have connections to multiple groups, which in the case of males may provide access to a higher number of females. These social network measures were calculated separately for the four field seasons. We z-transformed each measure for each period separately, and then calculated the individual averages across the periods where the individual was present.

When testing for the effect of the network measures on the number of offspring and the number of reproductive partners per individual, we considered the proportion of days an individual was observed during the different sampling periods as a potential confounding variable, because it may influence the social network and reproductive success measures and thereby drive positive relationships between them. However, the predicted effect of this variable on reproductive success measures is weak, as it is associated with the probability of sampling relatives of each individual. We found no significant correlation between this variable and the number of offspring or the number of reproductive partners. Therefore, we carried out the analyses both without and with the inclusion of this confounding variable, and found that most results remained unaffected (Table S7).

Female birth data collection. Birth data were collected *ad libitum* during daily observations. Dens and litters were actively searched around the time of birth, and presence of a litter was recorded only after finding pups. Older pups and juveniles were only included when the mother could be identified as one of the dogs from the existing database, and birth dates were estimated. Birth dates with an estimation error of more than ±15 days were excluded. We recorded the GPS position of the den location from which we calculated the distance between dens. We counted the number of pups in each litter, which provided information on the distribution of litter sizes (Table S12).

Additional information based on the literature

Differences in fertility between male wolves and dogs

An increase in reproductive efficiency was observed in numerous domesticated species (11). Domestication reduces breeding seasonality, leads to earlier sexual maturity (12) and increases the frequency of reproductive cycles, resulting in an increased reproduction rate (13, 14). An increased litter size compared with wild relatives is also frequently observed (14); for example, in dogs up to 17 pups per litter have been recorded (12). One hypothesis to explain this increase is the existence of a lower energetic expenditure for maternal care in domesticated species, allowing for a higher energy allocation in reproduction (14). As expected, wolves and dogs differ in their fertility, which generally is higher in dogs and not subject to seasonal changes. This increase in fertility can be seen as an adaptation to human-modified environments (15).

The increase in fertility in dogs results from several physiological and morphological changes relative to wolves. The comparison of the weight of testes of dogs and wolves raised in a similar environment, accounting for body weight differences, highlighted the presence of larger testes in dogs (42.3% increase compared with wolves during breeding season) (16). Another study found a similar relative weight of testes in free-ranging dogs compared with wild canids (17). However, the authors did not directly compare dog testes measurements with those of wolves, but instead used for the comparison published values from the red fox (*Vulpes vulpes*) and other non-*Canis* Carnivora (18). Apart from the testes weight comparison, both Haase (16) and Woodall & Johnstone (17) showed a positive relationship between epididymidis (*i.e.*, an organ for sperm storage) and body weight. Dogs had larger epididymidis compared with wolves, after accounting for body weight differences (16). Dogs were also shown to have an increased size of epididymidis compared with dingoes (19).

The expected functional consequence of increased size of testes and epididymidis is higher sperm production and storage (18). This is supported by a positive relationship between size and sperm production (volume and concentration) found based on 140 dogs of different breeds (20). An increase in sperm production with the size of testes and epididymidis could explain the higher sperm concentrations in dogs (150-300 million sperm/mL) compared with Mexican gray wolves (*Canis lupus baylei*) (<100 million sperm/mL) (21). Sperm quantity is a crucial factor in dog reproductive efficiency as it may determine the outcome of competition between different ejaculates (22). Sperm competition was thus proposed as an explanation for smaller relative testes and/or epididymidis size in monogamous wolves compared with polygamous dogs (16, 17, 19, 23).

Diet content and usage of human-derived food in FRDs

The diet of FRDs is opportunistic and the nature of consumed items varies greatly according to the availability of resources in their environment (24). Several studies on the feeding ecology of FRD populations showed that human-derived food predominated in their diet, for example in Poland (25) and Zimbabwe (26), where cereals given by farmers were the most abundant food identified. Similarly, in central India, up to 90% of the FRD diet was composed of human-derived food (27).

FRDs can also hunt, and a common food source is livestock, representing for example 74.3% of dog diet in northern India (28). In Zimbabwe, two of the three most consumed animal species identified in dog feces were domestic, but additional observations on feeding behavior indicated that most of these items were scavenged rather than preyed upon by dogs (26). Apart from livestock, other human commensals were found in FRD diets, in some cases as the most frequent item. For example, in south-eastern Brazil, a large part of the diet was composed of rats and mice, as well as invertebrates, especially hymenopterans, some of which are known to live in garbage environments (29).

Depending on the available food resources and the feeding habits of the group, FRD can also prey upon wild animals (12). For example, in the specific condition of a fenced hunting estate without any livestock or dumped garbage available, a pack of dogs was observed regularly feeding on wild ungulates in Spain (30). However, in the majority of studies it is not possible to disentangle the proportion of scavenged and hunted species, since in most cases behavioural

observations are lacking. A minor part of the FRD diet in Brazil, India, Italy, Poland and Zimbabwe was composed of wild animals (25-29, 31). In some cases, the proportion of wild animals in diet can be higher, for example in an FRD population from Mexico, it was estimated at 62.3% (32). However, none of the FRD populations studied relied solely on predation of wild animals to sustain themselves. A study of dogs from a nature reserve in South Australia showed that they specialized in wild animals, with mammals representing more than 98% of the ingested biomass (33). However, no distinction was made between FRD and dingoes among the 11 sampled individuals, and this result may therefore reflect the feeding behavior of dingoes which are ecologically different from FRDs.
Several studies have also highlighted the presence of vegetable material in the diet, for example in Mexico and Zimbabwe, where this type of food was the second and third most frequently found item in dog faeces, respectively (26, 32). Some populations even seem to specialize in plant food sources, for example vegetation was the most frequently found item in dog scats in south-eastern Brazil (29).

Mating system characteristics of FRDs based on observational data

Polygynandrous mating system. Behavioral observations suggest that polygynandry is common in FRDs, this mating system being reported in several FRD populations, for example in Italy (34, 35), in the USA (36) and India (37, 38).

Partner preferences. Behavioral observations indicate that both males and females show a preference for high-ranking partners (34, 37). Under confined conditions, strong preferences among female dogs for certain male partners were also found (39). In an urban environment, Daniels (36) identified a female preference for males ranging in a proximate area ("familiar males").

Allomaternal care. Due to the simultaneous presence of different breeding females in the same group of dogs, there is a potential for allomaternal care and litter sharing. For example, in North America, two lactating females and a single litter of ten pups were identified in the same pack, and the authors concluded that this may be a communally reared litter (40). Multiple breeding females were also reported in the same group in India, with allomaternal nursing consisting of milk sharing with pups from other litters (41). Pal et al. (42) also observed allomaternal care in Indian FRDs. Because allomaternal care was observed between related females, this behavior was interpreted under the inclusive fitness theory (42). In contrast, some breeding females in an FRD population in the USA were observed to emigrate temporarily to rear pups apart from the group, leading to a "pack splitting" (40). This behavior was also observed in Italy and interpreted as an avoidance of potential infanticides from other females of the pack (34).

Care from putative fathers. Males were observed to have more interactions with pups if they were supposedly the single mates of the pups' mothers, and their aggression toward vehicles, humans, and animals appearing near the pups was interpreted as protective behavior (43). One male also regurgitated food to the pups several times (43). Pup caring by males was also observed when males had mated with the mother of these pups, but paternity could not be tested and females were not reported to mate exclusively with these males (44). For these males, care included mainly play, protection, and sleeping in body contact, with food offering, regurgitation, and allogrooming occurring less commonly (44).

Seasonal monogamy. Variations in the polygynandrous mating system have been reported, usually based on behavioral observations during one oestrus period. For example, based on the observation that some females only mated with a single male and others with multiple males, including transient individuals, Pal (38) concluded that monogamy, polyandry, and polygyny are present in Indian free-ranging dogs. Additionally, opportunistic mating by transient males and forced mating was observed (38). Focal observations of six female FRDs revealed that each of them showed mating preferences toward particular males (43). Four of these six females were seen mating with only one male, which led to the conclusion that monogamy is an alternative

mating strategy in Indian dogs (43). Similarly, females who only mated with one male during the observation period were reported from an Italian FRD population (34). However, all these observations were made during one oestrus period, and while they suggest seasonal monogamy, there is no evidence for a long-term monogamy.

Sexual size dimorphism in FRDs

Modern domestic dog breeds exhibit sexual size dimorphism (SSD), with males being larger than females (45-48). Breed standards specifying that males should be taller than females likely have a reinforcing effect (45, 48). To our knowledge, only one study examined SSD in FRDs and indicated that males tended to be slightly larger than females in two packs where measurements were carried out (49).

Sutter et al. (48) used shoulder height data from at least three purebred individuals of each sex across 53 breeds and found that the average male-to-female ratio was 1.09, remaining consistent across breeds. Another study, however, reported variation in SSD in shoulder height across dog breeds (47). They measured 10 individuals of each sex from 74 breeds and found that SSD was more pronounced in larger breeds, while smaller breeds were nearly monomorphic (47). This pattern, where SSD increases with mean body mass (hyperallometry) in taxa where males are the larger sex, is known as Rensch's rule (50, cited by 51). Frynta et al. (47) argued that domestication did not have a marked effect on the magnitude of SSD in domestic dogs, since the male-female body mass ratio (Lovich-Gibbons ratio) in wolves (1.28) is comparable to the male-female body mass ratio in dog breeds of comparable sizes, e.g. hovawart (1.26), beauceron (1.25) and giant schnauzer (1.22) (data from 52).

While SSD is present in many wild canids as well (body mass: Macdonald & Sillero-Zubiri (53); shoulder height: Moehlman & Hofer (52); craniodental dimensions: Hatlauf et al. (54); Jojić et al. (55); Kennedy et al. (56); Milenvić et al. (57); Szuma (58, 59)), they remarkably do not follow Rensch's rule (47, 60, 61). Additionally, SSD is reported to vary geographically within species (e.g., tooth size in red fox: Szuma (59)). This is attributed to a lower selective advantage of larger male body size in wild canids. Indeed, canid characteristics, such as monogamous mating systems and group-hunting could reduce the importance of contest competition for male reproductive success (60, 61).

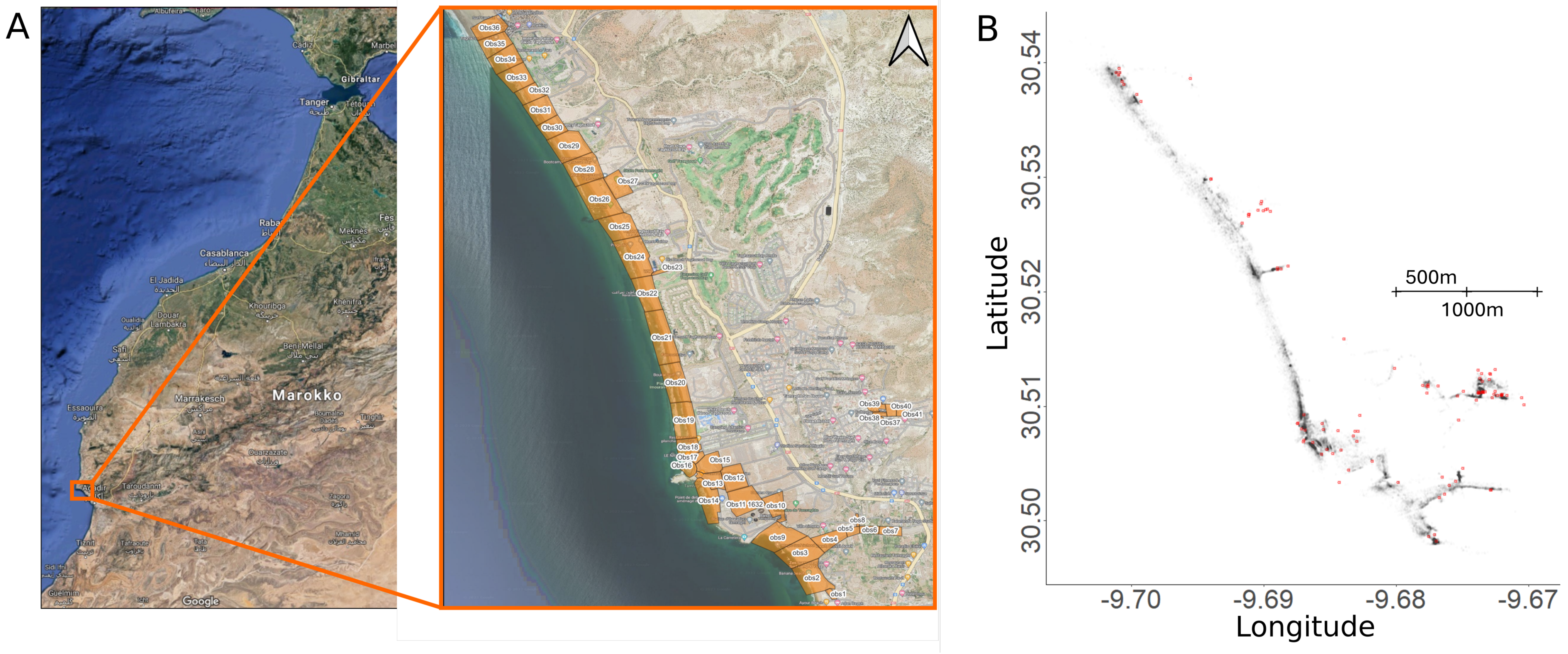

Fig. S1. Study area and distribution of dogs and dens. (A) Study area with the areas of regular data collection. (B) All GPS positions of individual dogs recorded between 2022 and 2024 (grey) and all GPS positions of dens recorded in the same period (red).

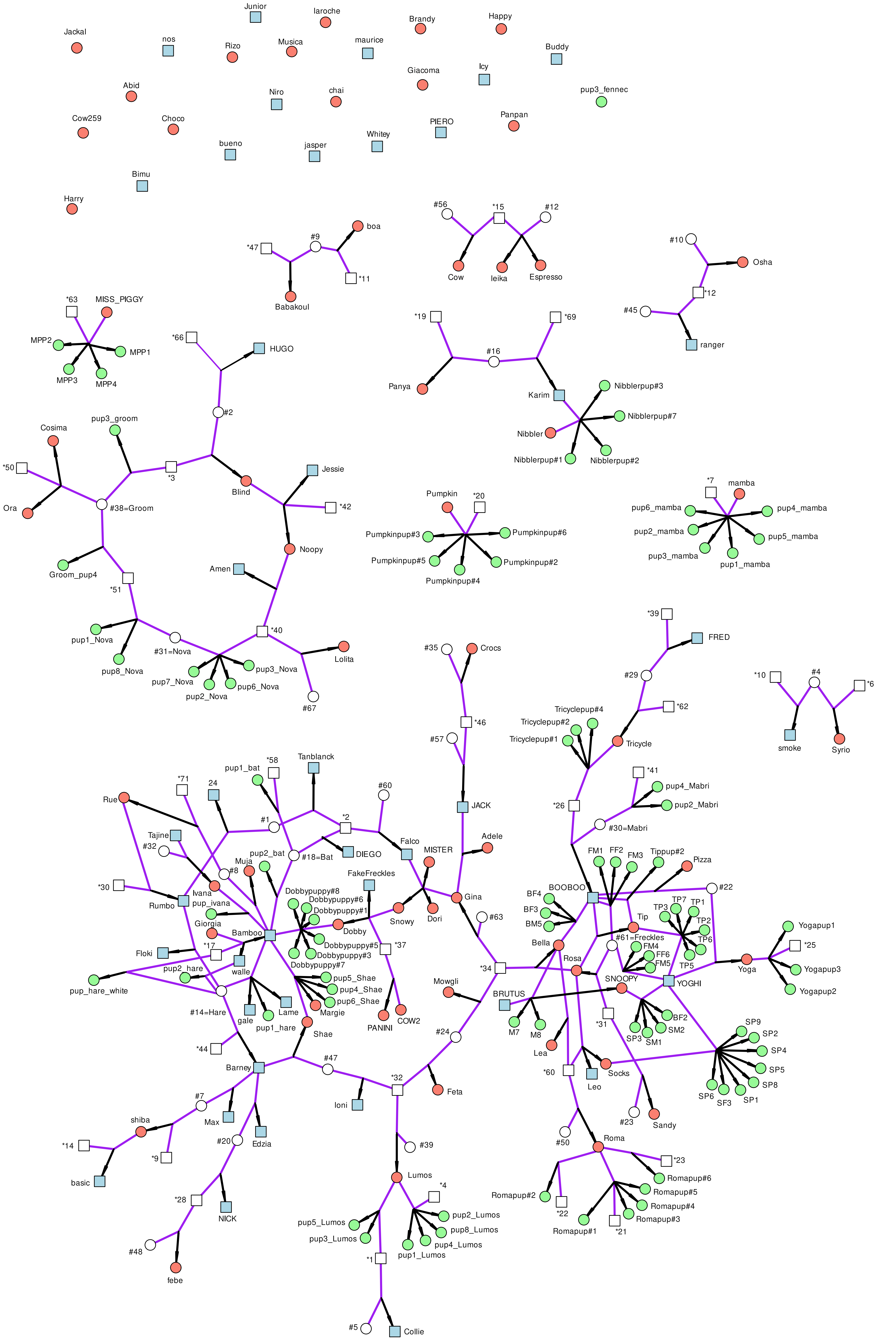

Fig. S2A. Genealogy of the Moroccan population reconstructed from genetic data. The networks show relationships at the parent-offspring and sibling level. This figure corresponds to this from Figure 1, but includes individual IDs.

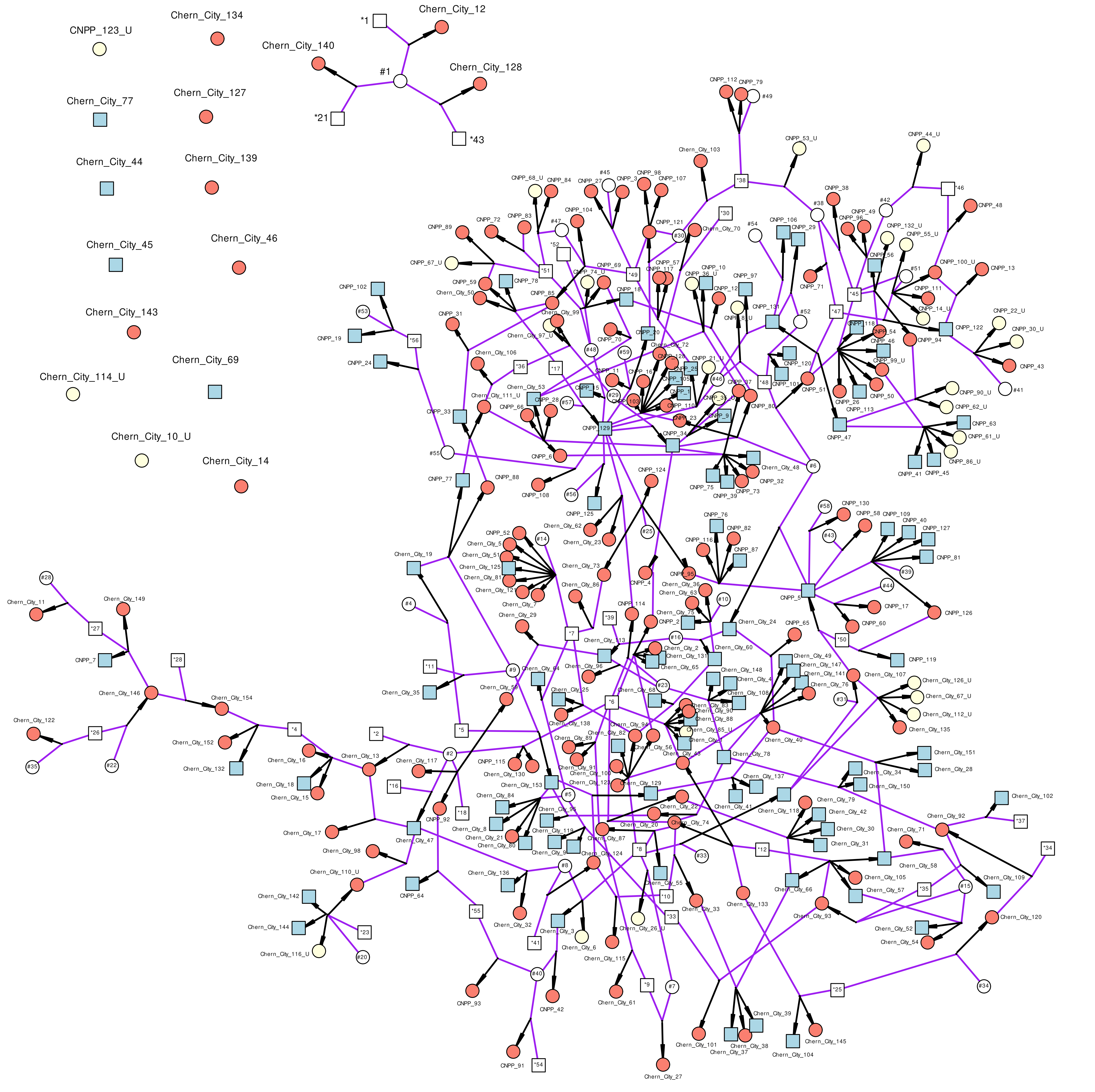

Fig. S2B. Genealogy of the Ukrainian population reconstructed from genetic data. The networks show relationships at the parent-offspring and sibling level. This figure corresponds to this from Figure 1, but includes individual IDs.

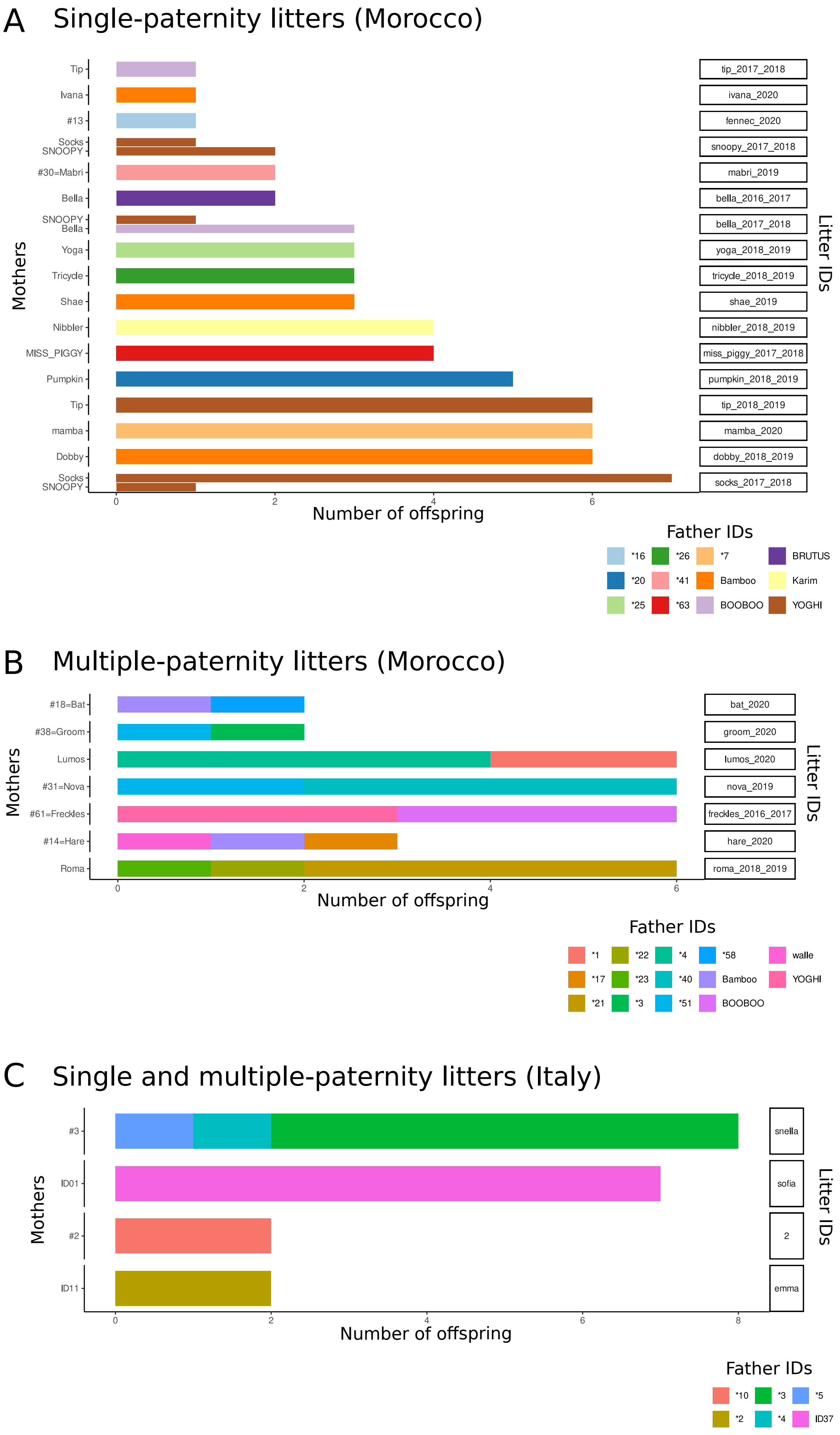

Fig. S3. Paternity in litters. (A) Litters with single paternity in the Moroccan population, (B) Litters with multiple paternity in the Moroccan population, (C) Litter paternity in the Italian population. Sampled fathers are marked with names, while fathers inferred in the genealogy reconstruction are marked with identification numbers. Three litters with two mothers shown in panel (A) represent a case of pup swapping between related mothers (see Figure S9).

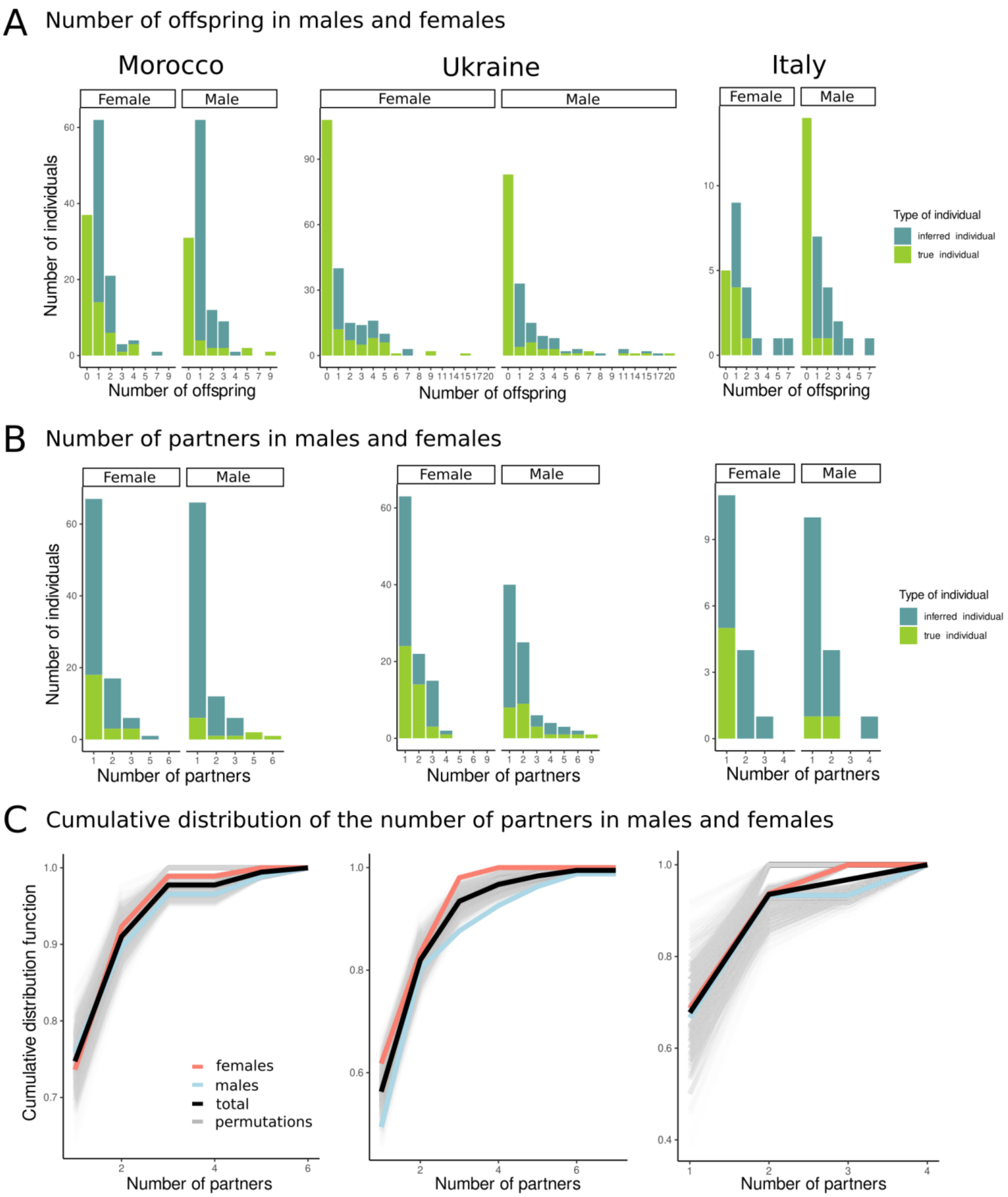

Fig. S4. (A) Distribution of the number of offspring for males and females, (B) Distribution of the number of reproductive partners for males and females, inferred based on the shared offspring; (C) Cumulative distribution of the number of partners for males and females in the three populations.

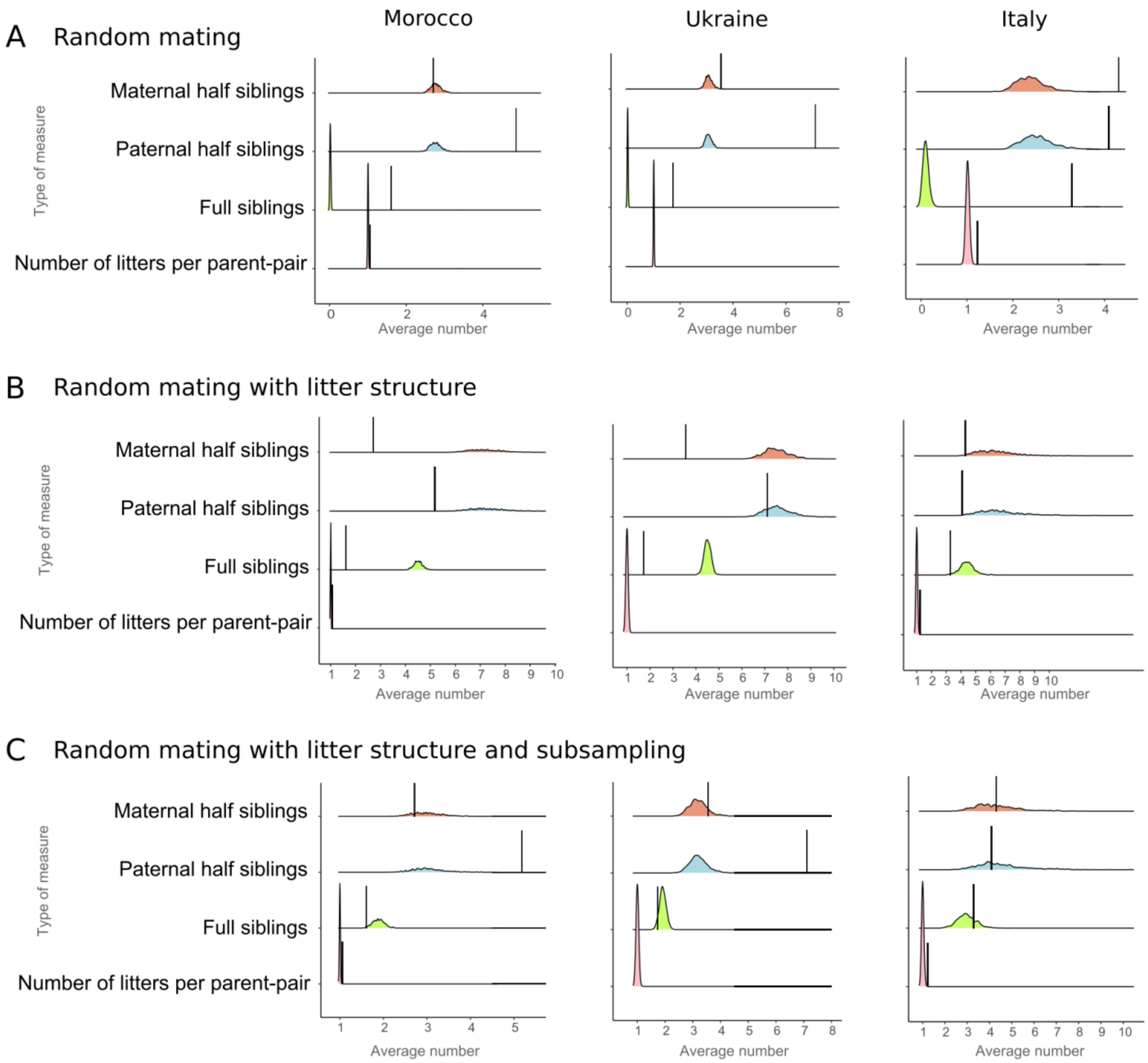

Fig. S5. (A) Numbers of full- and half-siblings and numbers of litters per parent pair in simulated populations with (A) fully random mating, (B) random mating with litter structure (see Methods), (C) subsampling from the randomly mating populations with litter structure. Three groups of simulations corresponded to the three study populations and were based on each population's parameters (see Methods). Within each group, 1000 simulations were run and the distribution of average values from each simulation are shown. The average values observed in the study populations are shown as vertical lines.

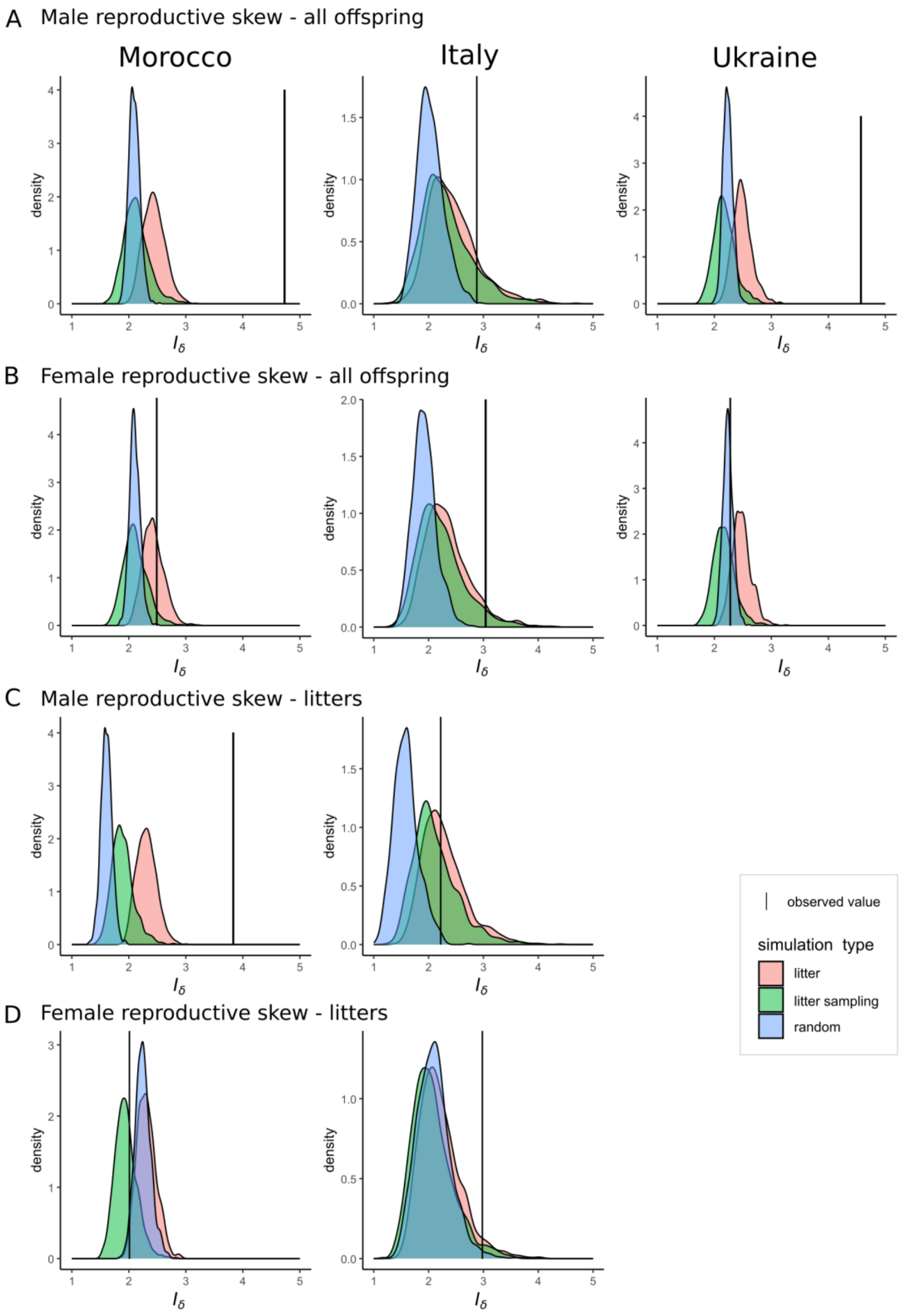

Fig. S6. Reproductive skew (*Iδ*) calculated for the study populations (black vertical line) and compared with the distribution of expected skew from the three simulation scenarios: random mating scenario (blue), random mating scenario with litter structure (red), random mating scenario with litter structure that match the sample sizes of the study populations (green). (A) Reproductive skew calculated for males and considering all offspring. (B) Reproductive skew calculated for females and considering all offspring. And reproductive skew calculated only for the parents of offspring sampled in litters compared with reproductive skew calculated only for the parents of simulated litters (individuals with at least one maternal half-sibling, or full-sibling, at the same generation), for males (C), and females (D).

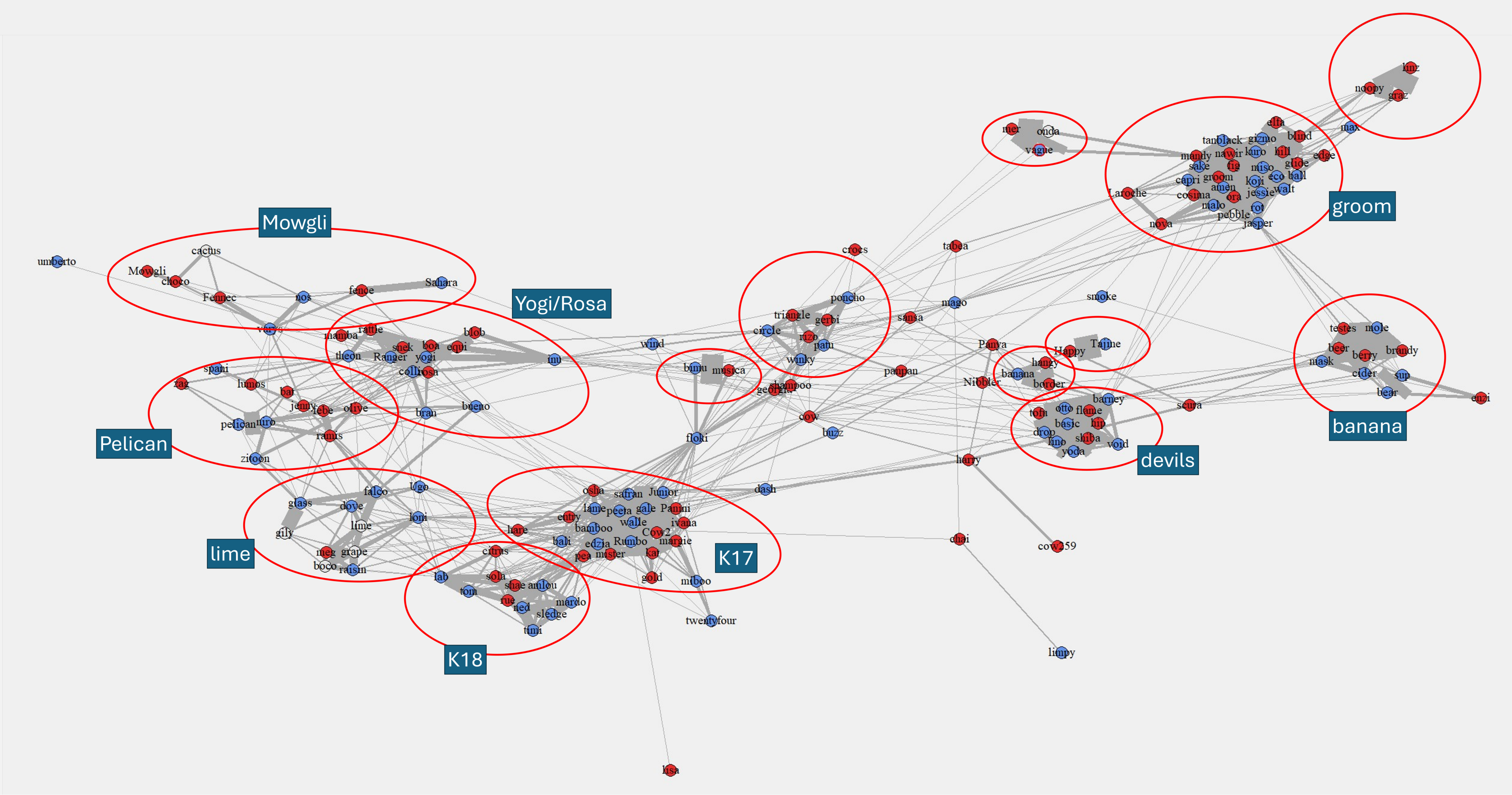

Fig. S7. Social network for the Moroccan population in season 2019-2020, with delimitation of social groups. Males are indicated with blue dots and females with red dots.

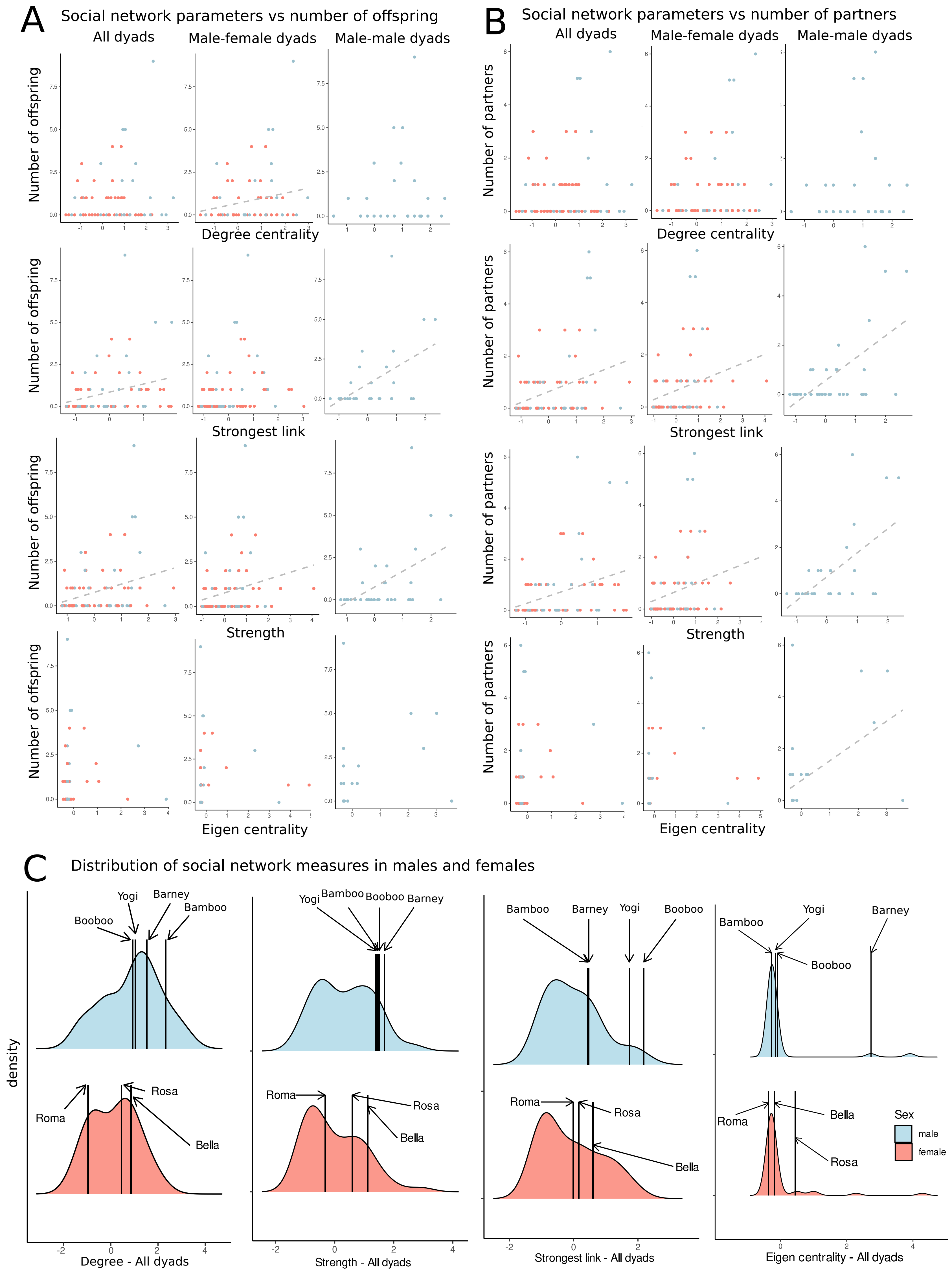

Fig. S8. The effect of social interactions, reflected in the position of an individual in the social network, on reproductive success in the Moroccan population. (A) Dependence of the number of offspring of individual dogs on the social network parameters *degree centrality*, *strength*, *strongest link* and *eigen-centrality*. (B) Dependence of the number of reproductive partners of individual dogs on the social network parameters. Plots for the parameter *betweenness* are not shown, because no significant correlation of this parameter with reproductive success or the number of reproductive partners was found. (C) Distribution of the social network parameters *strength, strongest link, degree centrality* and *eigen-centrality* values in males and females. These are the values from genotyped individuals only, which were extracted from the Z-transformed data for the entire social network. The values for the most successful breeders among the genotyped individuals are indicated with vertical lines.

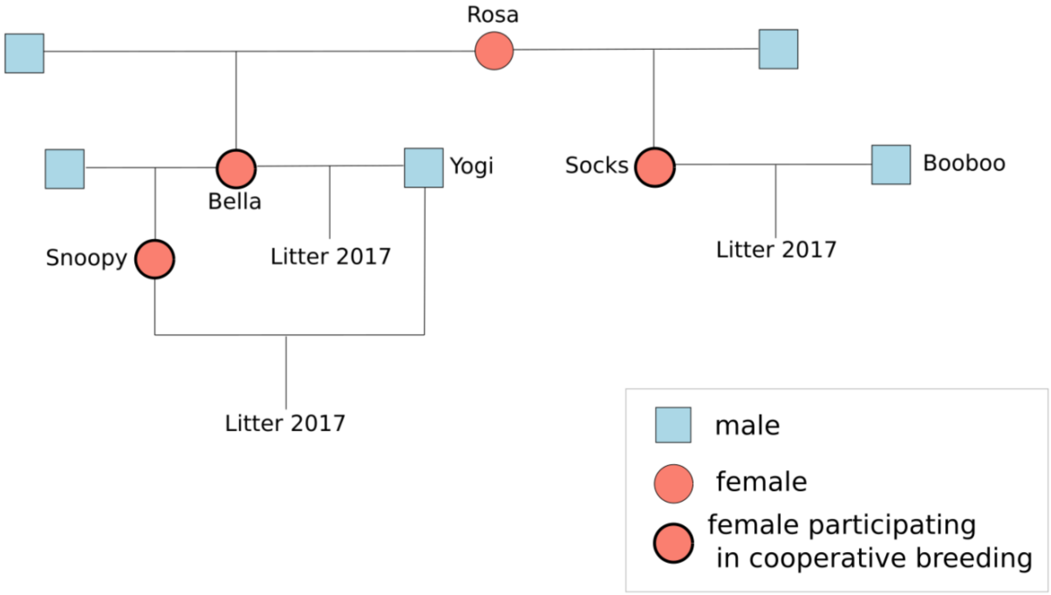

Fig. S9. Family tree showing kinship relationships of three females involved in cooperative breeding.

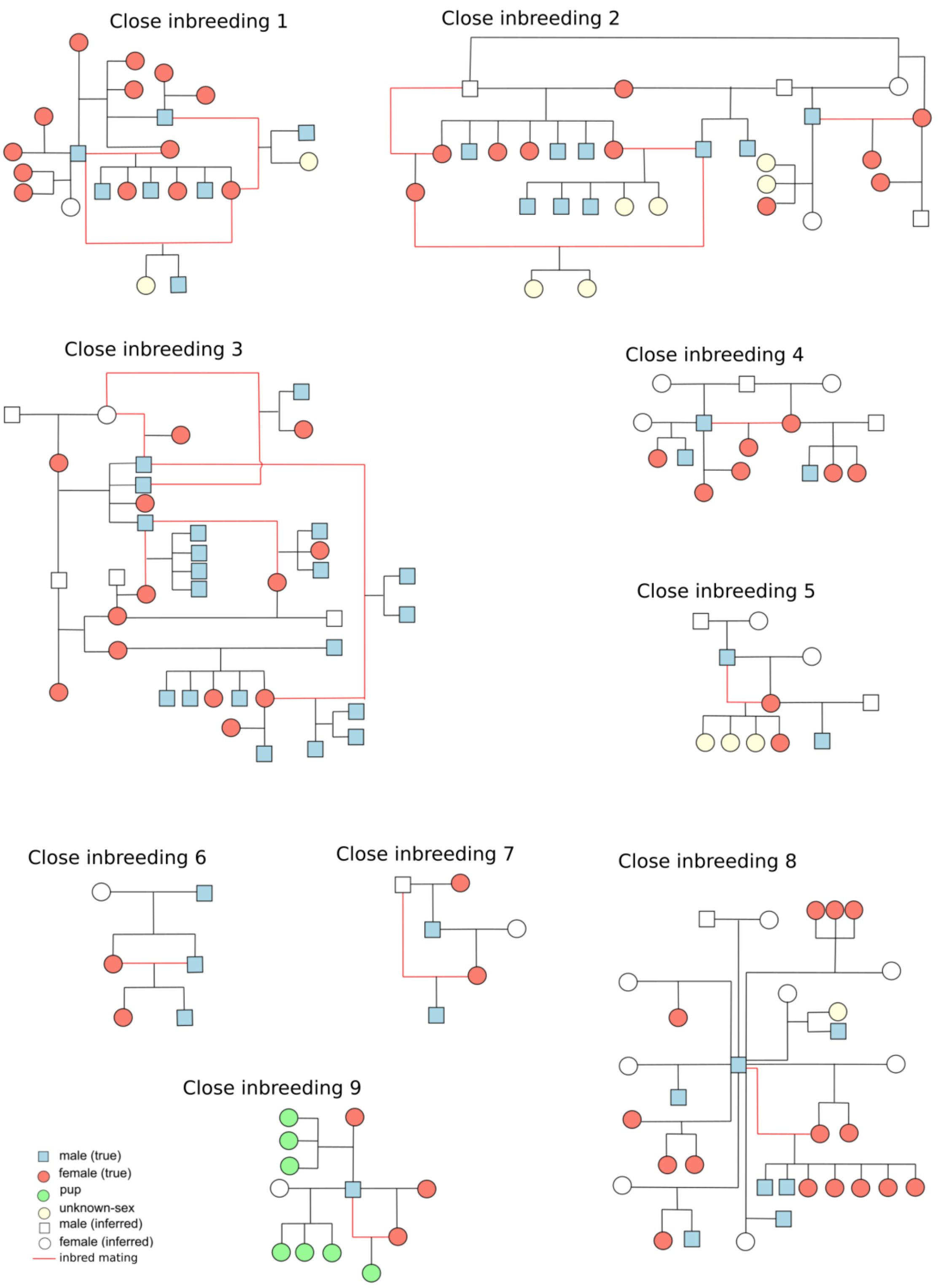

486

487 **Fig. S10.** Family trees with cases of inbred mating in the Moroccan and Ukrainian populations.
488 These trees are fragments of genealogies shown in Figure 1a. The close inbreeding cases are
489 numbered according to the order in Table S9.

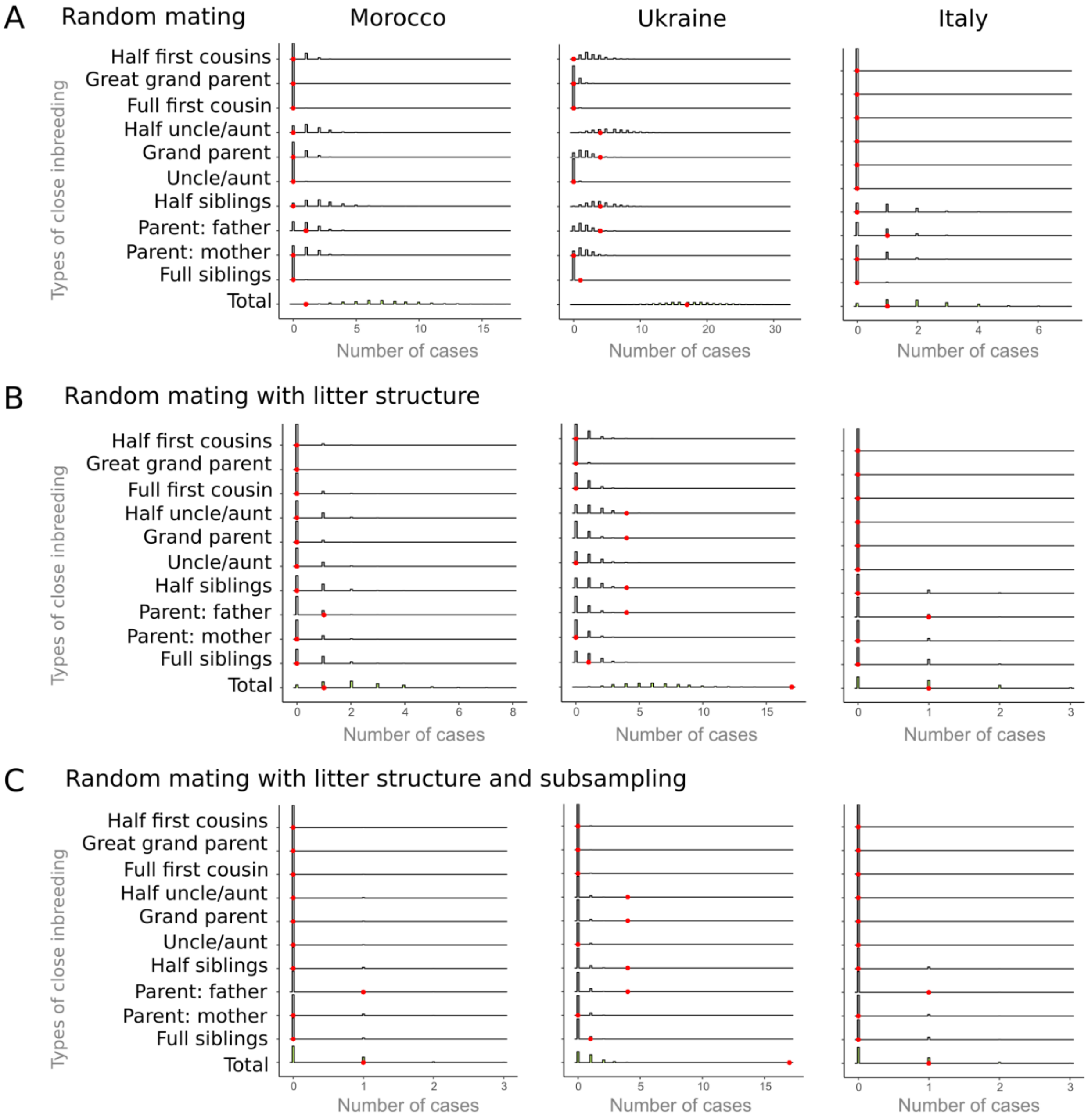

Fig. S11. Numbers of close inbreeding cases of different types in simulated populations. Three groups of simulations corresponded to the three study populations and were based on each population's parameters (see Methods). Within each group, 1000 simulations were run and the distribution of values from each simulation are shown. The values observed in the study populations are shown as red dots. Presented distributions are based on: (A) the simulated genealogies for the random mating scenario (B) the simulated genealogies for the random mating scenario with litter structure; (C) the subsampled genealogies for the random mating scenario with litter structure that match the sample sizes of the study populations.

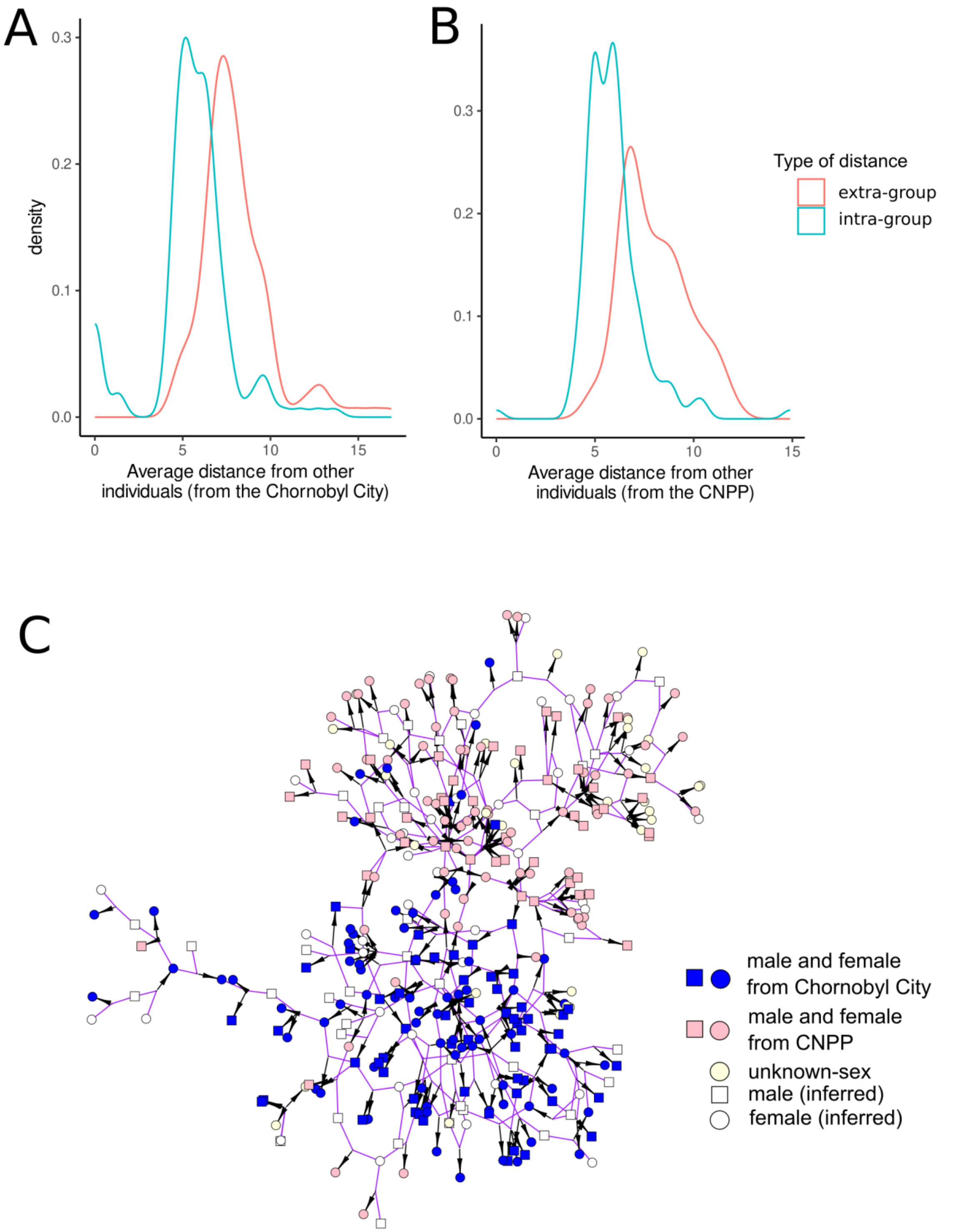

Fig. S12. Average individual distances along the pedigree to individuals from the other location in two parts of the Ukrainian population, which are 15 km apart. See Methods for the description of distance calculation. A. Distance of individuals from the Chornobyl Nuclear Power Plant to other individuals from the same location and to individuals from the Chornobyl City; B. Distance of individuals from the Chornobyl City to other individuals from the same location and to individuals from the Chornobyl Nuclear Power Plant; C. Genealogy of the Ukrainian dogs with individuals' sampling locations marked in different colours.

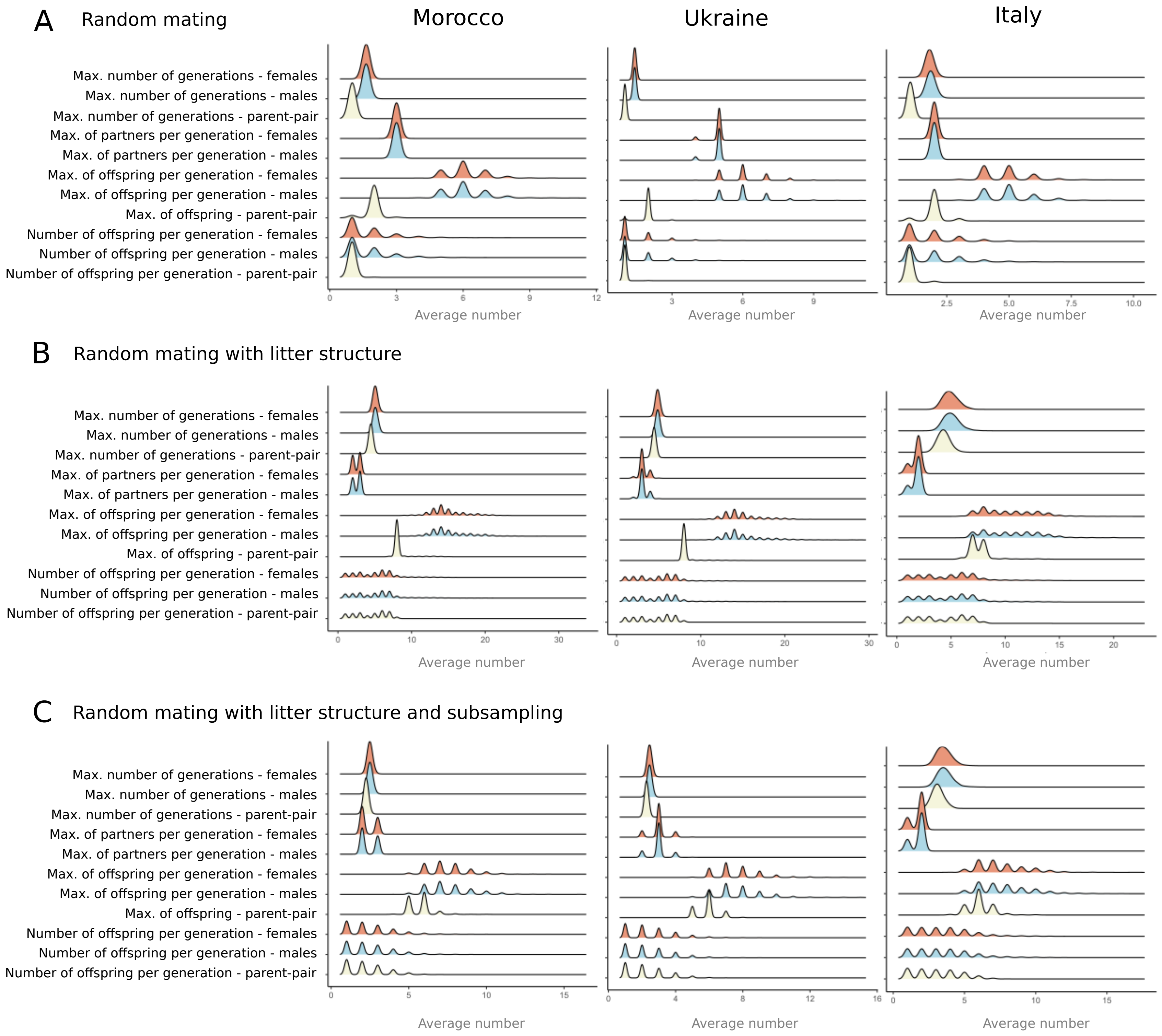

Fig. S13. Parameters of the simulations estimated per female, male and parent pair: the number of years an individual or the same pair reproduced, average number of offspring per year per simulation and the maximum number of offspring per year per simulation. In the simulations a year is assumed as the minimum breeding interval for females and a generation time in the simulations. The simulations modeled: (A) truly random mating, (B) random mating with litter structure (see Methods), (C) subsampling from the randomly mating populations with litter structure.

Table S1. Estimates of population size and population genetic parameters for the three populations studied.

Parameter	Morocco	Italy	Ukraine
Census population size	adults/subadults: 491<br>pups & juveniles: 132<br>all: 623	adults/subadults: 97	all: about 800
Population size in the genealogy	sampled: 196<br>inferred: 143<br>all: 339	sampled: 44<br>inferred: 24<br>all: 68	sampled: 286<br>inferred: 117<br>all: 403
Ne: random mating (CI95)	66 (49-95)	17 (9-34)	65 (48-94)
Ne: non-random mating (CI95)	71 (51-100)	32 (20-55)	58 (41-83)
N generations in the genealogy	4	3	6
F_{IS} (Colony)	−0.011	−0.177	0.035
F_{IS} (PLINK)	−0.016	−0.184	0.042
within-individual inbreeding coefficient	−0.018	−0.140	0.042
average PI-HAT (+/-SD)	0.049 (+/-0.090)	0.316 (+/-0.161)	0.037 (+/-0.090)
observed heterozygosity	0.331	0.574	0.340
expected heterozygosity	0.326	0.485	0.354

Table S2. The number of identified full siblings (FS), maternal half-siblings (MHS) and paternal half-siblings (PHS) per individual in the three populations. The difference between the number of MHS and PHS was tested using Wilcoxon sum rank test, with the P-values shown. (A) Measures for observed populations. In Morocco and Italy, we considered either all offspring of an individual, or one offspring per parent pair in litters. In Ukraine, the litter composition was unknown. (B) Measures for simulated populations. For the three population simulations, we considered the three different scenarios: random mating, random mating with litter structure, and random mating with litter structure with sampling of individuals. (C) Comparison between the observed measures considering all offspring and the most realistic simulation scenario with a litter structure and a sampling comparable with real sampling. Note that the standard deviations (SDs) reported in (A) are the SDs for each real population, while in (B) we report SDs from 1000 means obtained from the 1000 simulated populations.

**A. real populations**	Morocco (all offspring)	Morocco (one offspring per parent pair in litters)	Italy (all offspring)	Italy (one offspring per parent pair in litters)	Ukraine (all offspring)
N FS – mean +/-SD	1.61 +/-2.00	0.21 +/- 0.50	3.29 +/-3.08	1.63 +/- 2.43	1.73 +/- 1.84
N MHS – mean +/-SD	2.71 +/-2.38	1.10 +/- 1.51	4.31 +/-3.40	2.38 +/- 2.35	3.55 +/- 3.22
N PHS – mean +/-SD	5.18 +/-6.93	1.49 +/- 2.12	4.09 +/-2.82	2.31 +/- 2.19	7.11 +/- 6.09
Differentiation between maternal and paternal half-siblings	P = 0.369	P = 0.332	P = 0.857	P = 0.853	P = 2.6e-09

**B. simulated populations**	Morocco (random mating)	Morocco (litter structure)	Morocco (litter structure + sampling)	Italy (random mating)	Italy (litter structure)	Italy (litter structure + sampling)	Ukraine (random mating)	Ukraine (litter structure)	Ukraine (litter structure + sampling)
N FS – mean +/-SD	0.02 +/-0.01	4.51+/-0.17	1.89 +/-0.14	0.10 +/-0.05	4.42 +/-0.51	2.98 +/-0.43	0.02 +/-0.01	4.50 +/-0.14	1.91 +/-0.11
N MHS – mean +/-SD	2.78 +/-0.14	7.19 +/-0.65	3.00 +/-0.35	2.39 +/-0.30	6.39 +/-1.34	4.32 +/-1.00	3.08 +/-0.14	7.50 +/-0.56	3.19 +/-0.32
N PHS – mean +/-SD	2.76 +/-0.14	7.23 +/-0.64	3.03 +/-0.36	2.53 +/-0.34	6.67 +/-1.48	4.52 +/-1.09	3.08 +/-0.13	7.54 +/-0.57	3.20 +/-0.31
Differentiation between maternal and paternal half-siblings	P = 0.441	P = 0.169	P = 0.497	P < 2.2e-16	P = 6.0e-05	P = 2.8e-05	P = 0.041	P = 0.097	P = 0.112

**C. real vs simulated populations**	Morocco (all offspring)	Morocco simulation (litter structure + sampling)	Italy (all offspring)	Italy simulation (litter structure + sampling)	Ukraine (all offspring)	Ukraine simulation (litter structure + sampling)
N FS – mean +/-SD	1.61 +/-2.00	1.89 +/-0.14	3.29 +/-3.08	2.98 +/-0.43	1.73 +/- 1.84	1.91 +/-0.11
N MHS – mean +/-SD	2.71 +/-2.38	3.00 +/-0.35	4.31 +/-3.40	4.32 +/-1.00	3.55 +/- 3.22	3.19 +/-0.32
N PHS – mean +/-SD	5.18 +/-6.93	3.03 +/-0.36	4.09 +/-2.82	4.52 +/-1.09	7.11 +/- 6.09	3.20 +/-0.31
Differentiation between maternal and paternal half-siblings	P = 0.369	P = 0.497	P = 0.857	P = 2.8e-05	P = 2.6e-09	P = 0.112

Table S3. The percentage of adult females and males without offspring detected in each population.

	N females without offspring (total N females)	Percentage of females without offspring	N males without offspring (total N males)	Percentage of males without offspring
Morocco	37 (61)	60.7	31 (42)	73.8
Ukraine	108 (150)	72.0	83 (107)	77.6
Italy	5 (10)	50.0	14 (16)	87.5

Table S4. Opportunity for selection (*I*) and reproductive skew (I_{δ}) in males and females in the three populations. For Morocco and Italy, two numbers of offspring where considered: the total number of offspring of each individual ("All offspring") and a corrected number, with only one offspring per litter per parent pair ("one/pair"). For each population, the two measures were calculated for the sampled parents ("sampled"), and all the parents identified in the reconstructed pedigree, including individuals not present in the sample but inferred in COLONY ("Inferred + sampled"). The number of offspring identified in each dataset used for the calculation of *I* is indicated ("N offspring").

			I Females	I_{δ} Females	N offspring	I Males	I_{δ} Males	N offspring
Morocco	All offspring	Sampled	2.37	1.55	92	7.85	6.78	65
		Inferred + sampled	1.44	2.49	196	2.75	4.73	196
	One offspring/litter	Sampled	2.49	1.15	41	5.34	4.24	33
		Inferred + sampled	0.99	1.45	136	1.17	1.96	136
Ukraine		Sampled	4.88	2.12	142	7.73	4.84	122
		Inferred + sampled	2.35	2.28	286	3.67	4.57	286
Italy	All offspring	Sampled	3.12	2.06	13	12.63	5.44	9
		Inferred + sampled	1.67	3.04	45	2.36	2.88	45
	One offspring/litter	Sampled	1.36	0.47	6	8.41	2.33	3
		Inferred + sampled	1.28	2.38	32	2.07	2.31	32

Table S5. Dependence of the number of detected reproductive partners of an individual on the number of offspring and the sex. The table shows the results of type II F tests performed on generalized linear models with Poisson distribution.

	Sum Sq	Df	F value	P value
**Morocco**				
Number of offspring	114.50	1	388.79	2.94e-52
Sex	44.13	2	74.92	4.73e-26
Number of offspring: Sex	2.49	1	8.47	3.95e-03
**Ukraine**				
Number of offspring	233.69	1	332.58	1.81e-53
Sex	98.00	2	69.74	1.99e-26
Number of offspring: Sex	7.57	1	10.77	1.13e-03
**Italy**				
Number of offspring	13.57	1	24.51	8.81e-06
Sex	5.64	2	5.09	9.71e-03
Number of offspring: Sex	0.78	1	1.41	2.40e-01

Table S6. Pairs of individuals that produced multiple litters. The number of litters and the name of genotyped offspring is shown, as well as the number of other detected partners for the same year and other years. Some offspring were detected as adults rather than pups, and their year of birth was used to determine litters. The value of 0 does not imply the lack of other partners, it only shows that they were not detected in the dataset studied. Inferred fathers and mothers are labelled with * and #, respectively.

population	father	mother	N litters	offspring	N other partners the same year (male)	N other partners the same year (female)	N other partners other years (male)	N other partners other years (female)
Morocco	*37	Snowy	2	L1: Dobby	0	0	1	0
				L2: FakeFreckles	0	0	1	0
Morocco	Bamboo	Hare	3	L1: Gale	1	0	4	4
				L2: Lame	2	1	3	3
				L3: pup1_hare	2	2	3	2
Morocco	Brutus	Bella	2	L1: M7, M8	0	0	0	2
				L2: Snoopy	0	0	0	2
Italy	*3	Snella	2	L1: ID16, ID17, ID18, ID122, ID23	0	2	0	0
				L2: ID42, ID44	0	0	0	2
Italy	*6	#4	5	L1: ID24	0	0	0	0
				L2: ID30	0	0	0	0
				L3: ID32	0	0	0	0
				L4: ID41	0	0	0	0
				L5: ID47, ID48	0	0	0	0

Table S7. Statistical analysis of the dependence of the number of offspring and number of reproductive partners of an individual on two measures of the strength of social bonds: *strength*, i.e. the sum of weights of all connections of a given individual, *strongest link*, i.e. the highest weight of connection of an individual to any other population member, and one measure of social connectivity: *degree centrality*, i.e. an importance score based on the number of direct connections in the social network. Interactions of these explanatory variables with sex were assessed as well. The total number of offspring of each individual was considered ("all") as well as a corrected number, where only one offspring per litter per parent pair was assessed ("one/pair"). The significance of the predictor variables of generalized linear models with negative binomial distribution was tested using type II F tests. The table shows P-values, with significant values marked in bold. The stars denote the predictor variables that remained significant when applying alternative models that included the average number of days an individual was observed during the different sampling periods as a confounding variable. All interactions between the network measures and sex that were significant based on type II F tests remained significant when type III F tests were used instead.

Measure of reproductive success / dyads considered	N	Degree	Degree : Sex	Strength	Strength : Sex	Strongest link	Strongest link : Sex
N offspring (all)/ all dyads	78	0.4537	0.2018	**0.0062***	**0.0363***	**0.0068***	**0.0298***
N offspring (one/pair)/ all dyads	78	0.4612	0.3531	**0.0053***	0.2444	**0.0080***	0.1731
N offspring (all)/ FM dyads	78	**0.0362**	0.1535	**0.0366**	**0.0433***	0.1595	0.2515
N offspring (one/pair)/ FM dyads	78	0.0738	0.4401	**0.0152***	0.2475	**0.0234**	0.5996
N offspring (all)/ MM dyads	31	0.2950	NA	**0.0006***	NA	**0.0003***	NA
N offspring (one/pair)/ MM dyads	31	0.3209	NA	**0.0079***	NA	**0.0040***	NA
N offspring (all)/ FF dyads	47	0.2936	NA	0.4368	NA	0.1749	NA
N offspring (one/pair)/ FF dyads	47	0.3431	NA	0.4073	NA	0.1207	NA
N partners / all dyads	78	0.5853	0.2383	**0.0011***	0.0563	**0.0010***	**0.0468***
N partners / FM dyads	78	0.0637	0.1948	**0.0049***	**0.0332***	**0.0157***	0.3516
N partners / MM dyads	31	0.2892	NA	**0.0001***	NA	**0.0002***	NA
N partners / FF dyads	47	0.1754	NA	0.4146	NA	0.0979	NA

Table S8. Patterns of relatedness in social groups in FRD populations. Only social groups with at least three genotyped individuals were considered. Average pairwise relatedness is reported as PI-HAT and KING coefficients, with the KING coefficient multiplied by 2 to make it directly comparable with PI-HAT for positive values. Proportion of unrelated individuals is reported based on the PRIMUS result, considering the pairs classified as "unrelated" and "distant relatives" as unrelated for the purpose of this analysis. In the last column, the values in brackets provide the proportion of males and females among adult offspring found in a natal group. For the Moroccan population, the average values were calculated for all social groups considered as well as excluding the four groups for which only three individuals were sampled.

Population	Social group	Number of samples	Average PI-HAT	Average KING*2	Proportion of unrelated individuals	Proportion of parent pairs from the same group	Proportion of adult offspring found in a natal group (males/females)
Morocco	K17	28	0.066	0.025	0.21	0.74	0.80 (0.5/0.5)
Morocco	Yogi	16	0.090	0.010	0	0.50	0.50 (0.33/0.67)
Morocco	Groom	11	0.087	0.023	0.33	0.60	1 (0.5/0.5)
Morocco	Rosa	10	0.205	0.149	0.22	0.86	0.46 (0.29/0.71)
Morocco	Mowgli	6	0.038	0.015	1	no data	no data
Morocco	K18	6	0.073	0.031	0.17	0	0.50 (0/1)
Morocco	Feta	5	0.005	-0.067	1	no data	no data
Morocco	Devils	3	0.194	0.155	0.33	0.50	0.33 (1/0)
Morocco	Musica	3	0.020	-0.250	1	0	0.20 (1/0)
Morocco	Pelican	3	0.063	-0.153	0.33	no data	no data
Morocco	Poncho	3	0.036	0.000	1	no data	no data
Morocco	Average	-	0.080	-0.006	0.41	0.46	0.54 (0.52/0.48)
Morocco	Average (excl. last 4 groups)	-	0.081	0.027	0.42	0.54	0.65 (0.32/0.68)
Ukraine	ISF2	28	0.277	0.137	0	0.80	0.948 (0.54/0.46)
Ukraine	Pripyat	9	0.424	0.143	0	0.54	1 (0.41/0.59)
Ukraine	Semihody	14	0.122	0.018	0.071	0.19	0.875 (0.29/0.71)
Ukraine	Average	-	0.274	0.099	0.024	0.51	0.941 (0.41/0.59)

Table S9. Cases of close inbreeding observed in the study populations. If the parent pair was linked by more than one type of kinship relationship, only the closest one is reported. Pairwise relatedness coefficients (PI-HAT) are reported only for pairs of sampled parents. The column “group” refers to parent pairs co-occurring in the same family tree, as shown in Figure S10.

Population	Father	Mother	kinship relationship	PI-HAT	Group
Ukraine	Chern_City_53	CNPP_37	half siblings	0.48	1
Ukraine	CNPP_34	CNPP_37	father-daughter	0.57	1
Ukraine	CNPP_34	CNPP_6	father-daughter	0.50	1
Ukraine	*45	CNPP_54	father-daughter	n/a	2
Ukraine	CNPP_122	CNPP_100	half siblings	0.11	2
Ukraine	CNPP_47	CNPP_94	half uncle/aunt - half niece/nephew	0.12	2
Ukraine	CNPP_47	CNPP_99	half siblings	0.25	2
Ukraine	Chern_City_66	Chern_City_33	half uncle/aunt - half niece/nephew	0.15	3
Ukraine	Chern_City_66	Chern_City_22	half uncle/aunt - half niece/nephew	0.19	3
Ukraine	Chern_City_58	Chern_City_40	half uncle/aunt - half niece/nephew	0.16	3
Ukraine	Chern_City_58	#15	grandparent-grandchild	n/a	3
Ukraine	Chern_City_57	#15	grandparent-grandchild	n/a	3
Ukraine	Chern_City_47	Chern_City_13	half siblings	0.30	4
Ukraine	Chern_City_118	Chern_City_107	father-daughter	0.50	5
Ukraine	CNPP_77	CNPP_88	full siblings	0.54	6
Ukraine	*50	CNPP_126	grandparent-grandchild	n/a	7
Ukraine	CNPP_129	CNPP_23	grandparent-grandchild	0.28	8
Morocco	Booboo	Tip	father-daughter	0.55	9
Italy	*2	Emma	father-daughter	n/a	-

Table S10. Comparison of the mating system characteristics of FRDs and grey wolves. This table is based on Table 2 from Natoli et al. (1), which was modified and updated based on new information about the FRD populations studied. Information on FRDs based on genetic analyses is indicated with "(G)" and originates from Natoli et al. (1) and/or the current study. Information based on behavioral observations, based on 12, 34, 38, 40-43, 49, 62; is indicated with "(B)". Data on wolves are based on 60, 63-75. In wolves, all information except sexual size dimorphism is supported by genetic data.

Trait	Domestic dog	Grey wolf
Litter paternity	1–3 fathers (G)	Single
Multiple litters produced by the same parent pair	Less frequent but not uncommon (G), and suggested by alternative monogamous mating strategy in a minority of individuals (B*)	Frequent
Multiple breeding females within groups	Frequent (G, B)	Rare in stable populations but may be common in growing or heavily hunted populations
Maternal and paternal half-sibling relationships	Frequent (G)	Rare
Presence of unrelated individuals within groups	Frequent (G), and suggested by the presence of transient individuals within groups and recruitment of individuals from other groups (B)	Less frequent but not uncommon
Group affiliation of parents	Parent pairs from the same social group and those remaining in different groups before and after pups' birth are approximately equally frequent (G). Females were observed mating both with transient males and males from the same group (B)	The same group, with the exception of "sneaker" males breeding with females from different groups
Retention of adult offspring in natal groups	Frequent and similar for both sexes (G), but with a slight increase of female retention compared with males depending on the season (B)	Frequent
Dispersal among groups within the same area	Frequent (G, B)	Frequent
Close inbreeding	May be frequent in isolated populations; otherwise frequency consistent with that expected by chance (G)	Rare, but may occur in isolated populations; evidence for close inbreeding avoidance
Sexual size dimorphism	Moderate (B), with multiple equilibria in males (G)	Moderate

*Genetic data do not provide evidence for this.

Table S11. SNPs filtering steps used in the preparation of the genotyping data for kinship analyses in PRIMUS, CERVUS and COLONY. The exclusion criteria used to remove the SNPs from the analysed datasets are provided, e.g. ">10% missing data" means that all SNPs with more than 10% of missing data were removed from the dataset. Sample sizes obtained after each filtering step and used in a particular analysis are shown. SNP filtering was carried out in Plink1.9.
MAF – minor allele frequency; LD – linkage disequilibrium

Data processing step	Morocco	Ukraine	Italy
initial filtering	MAF<0.01; >10% missing data	MAF<0.01; >10% missing data	MAF<0.01; >20% missing data
N SNPs after filtering	138 693	125 419	140 061
IBD estimates and Primus analysis	138 693	125 419	140 061
LD pruning	$r^2 > 0.1$	$r^2 > 0.1$	$r^2 > 0.1$
pruning for Cervus and Colony analyses	MAF<0.48	MAF<0.49; >1% missing data	MAF<0.45
Colony analysis	1629	1671	1440
Cervus maternity and paternity analysis	1629	1671	2760
pruning for Cervus parent pair analysis	MAF<0.485	MAF<0.40; $r^2>0.09$	MAF<0.45; >2% missing data
Cervus parent pair analysis	1317	1198	1440

Table S12. Litter size distribution in Morocco used for the pedigree simulations attempting to replicate a realistic litter structure under random mating.

Litter size	Observed frequency	Modelled frequency
1	0.09	0.11
2	0.10	0.12
3	0.12	0.14
4	0.06	0.07
5	0.11	0.13
6	0.16	0.20
7	0.15	0.18
8	0.03	0.04
9	0.09	-
10	0.05	-
11	0.02	-
12	0.01	-
13	0.01	-

References

1. E. Natoli *et al*., Genetic inference of the mating system of free-ranging domestic dogs. *Behav. Ecol.* **32**, 646-656 (2021).
2. G. J. Spatola *et al*., The dogs of Chernobyl: Demographic insights into populations inhabiting the nuclear exclusion zone. *Sci. Adv*. **9**, eade2537 (2023).
3. E. Natoli *et al*., Data from: Genetic inference of the mating system of free-ranging domestic dogs. *Behav. Ecol.* doi: 10.5061/dryad.stqjq2c2q. (2021).
4. C. C. Chang *et al*., Second-generation PLINK: rising to the challenge of larger and richer datasets. *Gigascience* **4**, s13742-13015-10047-13748 (2015).
5. A. Manichaikul *et al*., Robust relationship inference in genome-wide association studies. *Bioinformatics* **26**, 2867-2873 (2010).
6. O. R. Jones, J. Wang, COLONY: a program for parentage and sibship inference from multilocus genotype data. *Mol. Ecol. Resour*. **10**, 551-555 (2010).
7. S. T. Kalinowski, M. L. Taper, T. C. Marshall, Revising how the computer program CERVUS accommodates genotyping error increases success in paternity assignment. *Mol. Ecol*. **16**, 1099-1106 (2007).
8. J. Staples *et al*., PRIMUS: rapid reconstruction of pedigrees from genome-wide estimates of identity by descent. *Am. J. Hum. Genet*. **95**, 553-564 (2014).
9. T. Pook, M. Schlather, H. Simianer, MoBPS-modular breeding program simulator. *G3* **10**, 1915-1918 (2020).
10. W. J. Hoppitt, D. R. Farine, Association indices for quantifying social relationships: how to deal with missing observations of individuals or groups. *Anim. Behav*. **136**, 227-238 (2018).
11. B. P. Setchell, Domestication and reproduction. *Anim. Reprod. Sci.* **28**, 195-202 (1992).
12. L. Boitani, P. Ciucci, Comparative social ecology of feral dogs and wolves. *Ethol. Ecol. Evol.* **7**, 49-72 (1995).
13. M. A. Zeder, The domestication of animals. *J. Anthropol. Res.* **68**, 161–190 (2012).
14. P. Cerrito, J. K. Spear, A milk-sharing economy allows placental mammals to overcome their metabolic limits. *Proc. Natl. Acad. Sci*. **119**, e2114674119 (2022).
15. K. Lord *et al*. Variation in reproductive traits of members of the genus *Canis* with special attention to the domestic dog (*Canis familiaris*). *Behav. Process.* **92**, 131-142 (2013).
16. E. Haase, Comparison of reproductive biological parameters in male wolves and domestic dogs. *Int. J. Mammal. Biol.* **65**, 257-270 (2000).
17. P. F. Woodall, I. P. Johnstone, Dimensions and allometry of testes, epididymides and spermatozoa in the domestic dog (*Canis familiaris*). *Reproduction* **82**, 603-609 (1988).
18. G. J. Kenagy, S. C. Trombulak, Size and function of mammalian testes in relation to body size. *J. Mammal.* **67**, 1-22 (1986).
19. P. F. Woodall, P. Pavlov, L. K. Tolley, Comparative Dimensions of Testes, Epididymides and Spermatozoa of Australian Dingoes (*Canis-familiaris-dingo*) and Domestic Dogs (*Canis-familiaris-familiaris*) Some Effects of Domestication. *Aust. J. Zool.* **41**, 133-140 (1993).
20. M. Tesi *et al*. Variables affecting semen quality and its relation to fertility in the dog: A retrospective study. *Theriogenology* **118**, 34-39 (2018).
21. P. Comizzoli *et al*. Current knowledge in the biology of gametes and embryos from Carnivora. *Theriogenology* **196**, 254-263 (2023).

22. G. A. Parker, “Sperm competition and the evolution of animal mating strategies.” in *Sperm competition and the evolution of animal mating systems*, R. L. Smith, Ed. (Academic Press, 1984), pp. 1-60.
23. A. Petersen *et al*. Characteristics of reproductive organs and estimates of reproductive potential in Scandinavian male grey wolves (*Canis lupus*). *Anim. Reprod. Sci.* **226**, 106693 (2021).
24. M. E. Gompper, *Free-ranging dogs and wildlife conservation* (Oxford University Press, 2014).
25. D. Krauze-Gryz, J. Gryz, Free-ranging domestic dogs (*Canis familiaris*) in Central Poland: density, penetration range and diet composition. *Pol. J. Ecol.* **62**, 183-193 (2014).
26. J. R. Butler, W. Y. Brown, J. T. Du Toit, Anthropogenic food subsidy to a commensal carnivore: the value and supply of human faeces in the diet of free-ranging dogs. *Animals* **8**, 67 (2018).
27. A. T. Vanak, M. E. Gompper, Dietary niche separation between sympatric free-ranging domestic dogs and Indian foxes in central India. *J. Mammal.* **90**, 1058-1065 (2009).
28. N. Mahar, B. Habib, S. A. Hussain, Do we need to unfriend a few friends? Free-ranging dogs affect wildlife and pastoralists in the Indian Trans-Himalaya. *Anim. Cons*. **27**, 53-64 (2024).
29. C. B. D. Campos *et al*. Diet of free-ranging cats and dogs in a suburban and rural environment, south-eastern Brazil. *J. Zool.* **273**, 14-20 (2007).
30. J. Duarte, F. J. García, J. E. Fa, Depredatory impact of free-roaming domestic dogs on Mediterranean deer in southern Spain: implications for human-wolf conflict. *Folia Zool.* **65**, 135-141 (2016).
31. D. Sogliani*, et al.* Citizen science and diet analysis shed light on dog-wildlife interactions in Italy. *Biodiv. Cons.* **32**, 4461-4479 (2023).
32. E. Carrasco-Román, *et al.* Contributions on the diet of free-ranging dogs (*Canis lupus familiaris*) in the Nevado de Toluca Flora and Fauna Protection Area, Estado de México, Mexico. *Rev. Mex. Biodivers.* **92** (2021).
33. J. C. Woinarski *et al.* The diet of the feral cat (*Felis catus*), red fox (*Vulpes vulpes*) and dog (*Canis familiaris*) over a three-year period at Witchelina Reserve, in arid South Australia. *Aust. Mammal.* **40**, 204-213 (2017).
34. S. Cafazzo et al. Social Variables Affecting Mate Preferences, Copulation and Reproductive Outcome in a Pack of Free-Ranging Dogs. *PLoS One* **9**, e98594 (2014).
35. D. W. MacDonald, G. M. Carr, “Variation in dog society: Between resource dispersion and social flux.” in *The Domestic Dog (2nd ed.),* J. Serpell, Ed. (*Cambridge University Press* , 1995), pp. 319–341.
36. T. J. Daniels, The social organization of free-ranging urban dogs. II. Estrous groups and the mating system. *Appl. Anim. Ethol.* **10**, 365–373 (1983).
37. B. Ghosh, D. K. Choudhuri, B. Pal, Some aspects of the sexual behaviour of stray dogs, *Canis familiaris*. *Appl. Anim. Behav. Sci.* **13**, 113–127 (1984).
38. S. K. Pal, Mating System of Free-Ranging Dogs (*Canis familiaris*). *Int. J. Zool.* **2011**, 1–10 (2011).
39. F. A. Beach, B. J. LeBoeuf, Coital behaviour in dogs. I. Preferential mating in the bitch. *Anim. Behav.* **15**, 546–558 (1967).
40. T. J. Daniels, M. Bekoff, Population and social biology of free-ranging dogs, *Canis familiaris. J. Mammal.* **70**, 754-762 (1989).
41. M. Paul, A. Bhadra, Selfish Pups: Weaning Conflict and Milk Theft in Free-Ranging Dogs. *PLoS One* **12**, e0170590 (2017).

42. S. K. Pal, S. Roy, B. Ghosh, Pup rearing: The role of mothers and allomothers in free-ranging domestic dogs. *Appl. Anim. Behav. Sci.* **234**, 105181 (2021).

43. S. K. Pal, Parental care in free-ranging dogs, *Canis familiaris*. *Appl. Anim. Behav. Sci.* **90**, 31–47 (2005).

44. M. Paul, A. Bhadra, The great Indian joint families of free-ranging dogs. *PLoS One* **13**, e0197328 (2018).

45. J. J. Carrasco *et al*. A pilot study of sexual dimorphism in the head morphology of domestic dogs. *J. Vet. Behav.* **9**, 43–46 (2014).

46. K. Chase *et al*. Genetic basis for systems of skeletal quantitative traits: Principal component analysis of the canid skeleton. *Proc. Natl. Acad. Sci*. **99**, 9930–9935 (2002).

47. D. Frynta *et al*. Allometry of Sexual Size Dimorphism in Domestic Dog. *PLoS One* **7**, e46125 (2012).

48. N. B. Sutter, Morphometrics within dog breeds are highly reproducible and dispute Rensch's rule. *Mammal. Genome* **19**, 713–723 (2008).

49. R. Bonanni *et al*. Age-graded dominance hierarchies and social tolerance in packs of free-ranging dogs. *Behav. Ecol.* **28**, 1004–1020 (2017).

50. B. Rensch, Die Abhängigkeit der relativen Sexualdifferenz von der Körpergröße. *Bonner Zoologische Beiträge : Herausgeber: Zoologisches Forschungsinstitut Und Museum Alexander Koenig, Bonn* **1**, 58—69 (1950).

51. E. Abouheif, D. J. Fairbairn, A Comparative Analysis of Allometry for Sexual Size Dimorphism: Assessing Rensch's Rule. *Am. Nat.* **149**, 540–562 (1997).

52. P. D. Moehlman, H. Hofer, "Cooperative Breeding, Reproductive Suppression, and Body Mass in Canids." in *Cooperative Breeding in Mammals.* J. A. French & N. G. Solomon, Eds. (Cambridge University Press, 1997), pp. 76–128.

53. D. W. Macdonald, C. Sillero-Zubiri, *The Biology and Conservation of Wild Canids*. (Oxford University Press, 2004).

54. J. Hatlauf *et al*. The canine counts! Significance of a craniodental measure to describe sexual dimorphism in canids: Golden jackals (*Canis aureus*) and African wolves (*Canis lupaster*). *Mammal. Biol.* **101**, 871–879 (2021).

55. V. Jojić, J. Porobić, D. Ćirović, Cranial variability of the Serbian red fox. *Zool. Anz.* **267**, 41–48 (2017).

56. M. L. Kennedy *et al*. An assessment of geographic variation in sexual size dimorphism in the coyote (*Canis latrans*). *Mammalia* **67**, 411–418 (2003).

57. M. Milenvić *et al.* Skull variation in Dinaric-Balkan and Carpathian gray wolf populations revealed by geometric morphometric approaches. *J. Mammal.* **91**, 376–386 (2010).

58. E. Szuma, Variation and correlation patterns in the dentition of the red fox from Poland. *Ann. Zool. Fenn*. **37** (2000).

59. E. Szuma, Geography of sexual dimorphism in the tooth size of the red fox *Vulpes vulpes* (Mammalia, Carnivora). *J. Zool. Syst. Evol. Res*. **46**, 73–81 (2008).

60. C. J. Bidau, P. A. Martinez, Sexual size dimorphism and Rensch's rule in Canidae. *Biol. J. Linn. Soc.* **119**, 816-830 (2016).

61. P. J. Johnson *et al*. Rensching cats and dogs: Feeding ecology and fecundity trends explain variation in the allometry of sexual size dimorphism. *R. Soc. Open Sci.* **4**, 170453 (2017).

62. S. K. Pal, B. Gosh, S. Roy, Dispersal behaviour of free-ranging dogs (*Canis familiaris*) in relation to age, sex, season and dispersal distance. *Appl. Anim. Behav. Sci.* **61**, 123-132 (1998).
63. N. Lehman *et al*. A study of the genetic relationships within and among wolf packs using DNA fingerprinting and mitochondrial DNA. *Behav. Ecol. Sociobiol*. **30**, 83-94 (1992).
64. T. L. Hillis, F. F. Mallory, Sexual dimorphism in wolves (*Canis lupus)* of the Keewatin District, Northwest territories, Canada. *Can. J. Zool.* **74**, 721-725 (1996).
65. D. Smith *et al*. Is incest common in gray wolf packs?. *Behav. Ecol*. **8**, 384-391 (1997).
66. W. Jędrzejewski *et al.* Genetic diversity and relatedness within packs in an intensely hunted population of wolves *Canis lupus*. *Acta Theriol*. **50**, 3-22 (2005).
67. B. M. vonHoldt *et al*. The genealogy and genetic viability of reintroduced Yellowstone grey wolves. *Mol. Ecol*. **17**, 252-274 (2008).
68. R. Caniglia *et al.* Noninvasive sampling and genetic variability, pack structure, and dynamics in an expanding wolf population. *J. Mammal.* **95**, 41-59 (2014).
69. I. Trbojević, D. Ćirović, Sexual dimorphism and population differentiation of the wolf (*Canis lupus*) based on morphometry in the Central Balkans. *North-West. J. Zool.* **12**.2 (2016).
70. D. E. Ausband, Multiple breeding individuals within groups in a social carnivore. *J. Mammal*. **99**, 836-844 (2018).
71. D. E. Ausband, Pair bonds, reproductive success, and rise of alternate mating strategies in a social carnivore. *Behav. Ecol*. **30**, 1618-1623 (2019).
72. V. Sidorovich, I. Rotenko, Reproduction biology in grey wolves *Canis lupus* in Belarus: common beliefs versus reality. *Chatyry Chverci,* (2019).
73. L. D. Mech, R. McIntyre. An observation of incest avoidance in Gray Wolf (*Canis lupus*). *Can. Field-Nat*. **137**, 232-234 (2024).
74. L. D. Mech, Plural breeding in Gray Wolf (*Canis lupus*) packs: how often? *Can. Field-Nat.* **138**, 58-62 (2024).
75. C. Pacheco *et al.* Relatedness-based mate choice and female philopatry: inbreeding trends of wolf packs in a human-dominated landscape. *Hered*. **132**, 211-220 (2024).

Additional resource (separate file). SNP genotypes from the Moroccan dogs used to reconstruct the genealogy. Files in Plink format (.ped, .map) for 163,594 autosomal SNPs for 196 free-ranging Moroccan dogs (available in the Figshare repository).

Dataset S1 (separate file). Reconstructed pedigrees including all the individuals sampled in the three populations. The name of the father and mother is indicated as well as the number of offspring and partners. For Morocco and Italy, three different offspring numbers for each individual correspond to (i) the total number of offspring, (ii) a corrected measure considering only one offspring per parent pair in litters, and (iii) a measure excluding individuals sampled as pups (available in the Figshare repository).

Dataset S2 (separate file). Social network data for the Moroccan population. These individual social network measures are the average of measures collected from 2017 to 2020. For each year and every individual, values were z-transformed. The dataset also includes information about sex of individuals and the reproductive success data inferred from the genealogy (the number of offspring and the number of reproductive partners) (available in the Figshare repository).

Dataset S3 (separate file). R scripts used for the data analysis. R markdown file shortly describing and compiling the different analyses performed on pedigrees: data extraction, computation and comparison of the number of offspring and partners, tree parentage figures, identification of close inbreeding cases, sex-biased dispersal in Ukraine, simulations of random mating, social network analysis, computation of the average number of full and half-siblings, code to run pedigree simulation, and analyses of simulation outputs (close inbreeding cases, pedigree statistics, subsampling) (available in the Figshare repository).
